# Contrasting pH optima of β-lactamases CTX-M and CMY influence *Escherichia coli* fitness and resistance ecology

**DOI:** 10.1128/aem.01775-25

**Published:** 2025-12-29

**Authors:** Mikkel Anbo, Saria Otani, Mirena Ivanova, Hanne Nørgaard Nielsen, Jacob Dyring Jensen, Christina Aaby Svendsen, Chengfang Pang, Frank M. Aarestrup

**Affiliations:** 1 DTU National Food Institute, Technical University of Denmark, Lyngby, Denmark; Centers for Disease Control and Prevention, Atlanta, Georgia, USA

**Keywords:** CMY-2, CTX-M-15, microbial ecology, pH, selection, β-lactamases, antimicrobial resistance

## Abstract

Antimicrobial resistance is one of the largest and most pressing global health threats. This is not only a huge burden on the global economy but also a growing threat to animal, environmental, plant, and human health, and new strategies are needed to avoid resistance and improve treatment. Novel antimicrobial resistance genes are normally first detected once they cause problems in clinical infections, and we have limited knowledge on their evolutionary trajectories. Current antimicrobial susceptibility testing and research have a limited focus on key environmental factors in pathogen-reservoir-host interactions, possibly leading to inaccurate results that do not reflect the *in vivo* conditions. Focusing on differences in pH, we determined the MIC of a panel of isogenic strains expressing CTX-M-15 and CMY-2 β-lactamases. We found that pH has a large impact on the activity of β-lactamases, and treatment of these resistant isolates could be possible if the pH of the environment is modified. We verified this using enzyme kinetics, co-cultures, and growth experiments, suggesting that exposure to different environmental conditions may lead to distinct evolutionary trajectories for specific β-lactamases. Exploring the effect of different temperatures, we also observed a differential effect of avian and mammal host temperatures. Environmental factors such as pH and temperature may have a large unnoticed effect on antimicrobial resistance, and we might use this knowledge to renew and extend the use of old antibiotics for certain infections.

**IMPORTANCE:**

Antimicrobial resistance is a huge burden to global health and economy. We need new options for avoiding selection of resistance and improved treatment. Overlooked aspect: current susceptibility testing does not take pH into account. With this study, we show that pH and temperature can have large and contrasting effects on the activity (and therefore MIC) of specific β-lactamases. This might help to explain the phenomenon of bacteria often harboring multiple β-lactamases seemingly with the same function as well as be utilized to enable treatment of genotypically resistant strains under very specific conditions, that is, treatment of CTX-M-15, the most prevalent ESBL in healthcare, under alkaline conditions.

## INTRODUCTION

Antimicrobial resistance (AMR) is responsible for millions of deaths every year and threatens global health, food production, and economy (1–5). Developing novel antimicrobials will not solve this problem; efforts must also focus on reducing the emergence and transmission of known and novel antimicrobial resistance genes (ARGs) (6). While antimicrobial consumption is a main driver for AMR, recent studies have shown that additional factors, including hygiene, co-selection, environmental conditions, and modes of use, also contribute to AMR emergence and dissemination (7–10).

Emergence and evolution of AMR is a complex process often involving stealthy transmission of novel ARGs between multiple bacterial taxa and mobile elements before their eventual identification in human, animal, or plant pathogens (11–13). Different niches involved in the early emergence and evolution of ARGs may have different environmental conditions, possibly leading to different resistance evolutionary trajectories. Recognizing these differences emphasizes the need for an integrated One Health approach (14). These different niches, which may vary in their physiochemical properties and contents such as antibiotics, pH, redox potential, temperature, hydration, metal concentrations, and oxygen pressure, create selective compartments (15) favoring certain ARGs or ARG variants. There has been much focus on the effect of antibiotic concentration (sub-lethal, selection window hypothesis) in terms of selection pressure (16–19) to reduce the emergence of AMR; however, environmental selection compartments (e.g., pH, temperature, and oxygen) have scarcely been considered, with only a few studies addressing these factors (20–25).

Clinical microbiology and AMR research have long prioritized standardization of antimicrobial susceptibility testing (AST), potentially overlooking the importance of environmental heterogeneity in different niches, hosts, and during infections (25, 26). AST is typically done in Cation-ion Adjusted Mueller Hinton broth (CAMHB), at pH 7.2–7.4 and 35°C ± 1°C, which has been widely adopted (ISO 20776-1 [2019], CLSI, EUCAST) as it allows for the growth of a wide range of pathogens. For practical reasons, such simplified technology may lead to inaccurate or uncertain AST (27) and biased assessment of the ecology and evolution of different ARGs.

Environmental factors such as temperature, oxygen pressure, and pH can largely influence bacterial growth in natural reservoirs and infections. Within the human body, and especially in infection-prone sites, there can be large variations in pH conditions between tissues and fluids (28). One example includes the human gastrointestinal (GI) tract (29), which ranges from the highly acidic stomach to the colon, where the pH fluctuates between acidic and weakly alkaline (30–33). The bladder can vary as much as between pH 4.5 and 8 (28, 34) and may even change due to infection (35). Yang et al. (22) and Kincses et al. (23) showed that pH plays a significant role in the efficacy of antibiotic treatment of several major susceptible and resistant uropathogens, and the urinary tract pH can be easily modified with existing treatments to potentially improve treatment. Despite evidence that pH influences antibiotic susceptibility in clinical isolates (20, 22–24, 36, 37), how pH modulates phenotypic resistance through its effects on individual ARGs, β-lactamase activity, and enzymes, and bacterial fitness remains largely unexplored. Recently, Ortiz-Miravalles et al. (25) described a differential effect of oxygen pressure on isogenic strains containing different ARGs, further suggesting that the classical MIC testing is limited.

The β-lactams, including penicillins and cephalosporins, are the most widely used class of antibiotics globally (38, 39). Third-generation cephalosporin (3GC) resistant *Escherichia coli* are considered to be one of the most critically important bacterial pathogens globally both in terms of mortality and years of life lost due to infections (40–43). These resistant *E. coli* can be transmitted between humans and animals and are thus continuously exposed to multiple environments (44–47). Some of the most prevalent genes causing resistance to 3GC are from the CTX-M group (48–53) and specifically CTX-M-15, as well as the CMY group, especially CMY-2 (54, 55). Many bacterial isolates harbor multiple seemingly redundant ARGs conferring resistance to the same and/or related antimicrobial agents (56–60). This might simply be due to multiple random plasmid acquisitions with limited selection pressure against this redundancy due to the low cost of carrying ARGs (61, 62). However, to our knowledge, this has never been proven. Another possible explanation for this redundancy may be that multiple β-lactamases can act synergistically and provide fitness to resistant organisms in environments with heterogeneous presence of different antibiotics (i.e., between different patients or niches).

In this study, we investigated how pH influenced 3GC resistance mechanisms in *E. coli*. Using constructed isogenic strains, expressing either CTX-M-15, CMY-2, or both enzymes, we showed a differential effect of high versus low pH depending on which gene was present and potentially identified conditions where isolates normally phenotypic resistant could be susceptible. This was then confirmed with targeted enzymatic kinetic assays. Using co-culturing and competition assays under different and fluctuating pH conditions, we showed a selective landscape where susceptible isolates would prevail. Our study demonstrates the importance of environmental conditions in shaping treatment efficacy, resistance ecology, and evolution of AMR, including a possible novel explanation for the presence of seemingly redundant genes. Future AST frameworks may integrate environmental heterogeneity practically by (i) the inclusion of optional adjunct panels testing defined pH conditions relevant to specific infection sites; (ii) reporting “environmental susceptibility profiles” for research, surveillance, and special clinical cases; and (iii) incorporating mechanistic modifiers (e.g., pH-dependent MIC shifts) into epidemiological cutoff value discussions when biologically relevant.

## RESULTS

### MIC profiles of CTX-M-15 and CMY-2 strains reveal opposing pH-dependent resistance patterns

**Fig 1:**
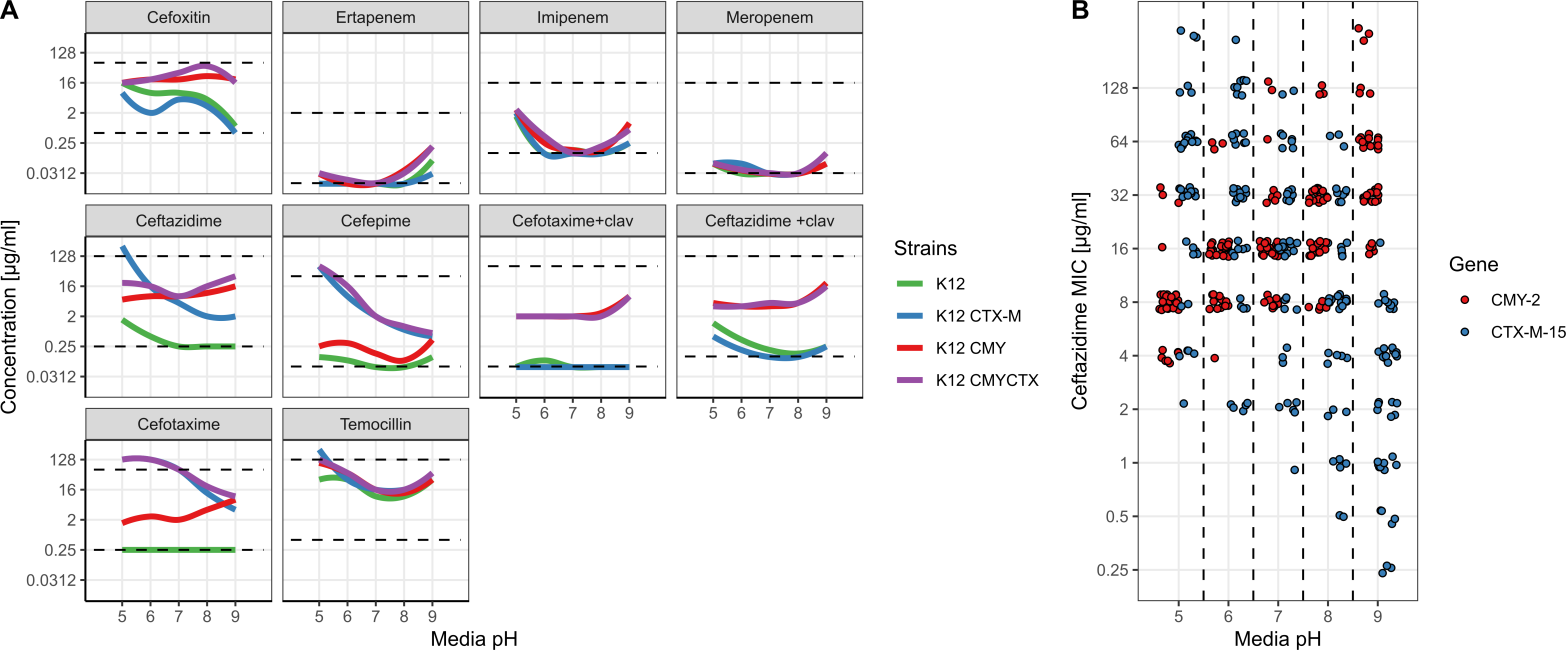
Media pH (abscissa) differentially affects β-lactamase resistance (MIC, ordinate). (**A**) MIC of K12 (wt), K12 CTX-M, K12 CMY, or K12 CMYCTX to different β-lactam compounds at different pH values. Each panel shows the MIC of strains shown at different pH levels, and the β-lactam compound is shown above each panel. The dashed lines indicate the minimum and maximum antibiotic concentration in the MIC panels. Panels denoted “+clav” are supplemented with 4 µg/mL clavulanic acid, a β-lactamase inhibitor. (**B**) Ceftazidime MIC distributions of 27 different wild-type clinical *E. coli* isolates with either β-lactamase CTX-M-15 or CMY-2 assayed at different pHs.

To determine if pH has an effect on the activity of β-lactamase-mediated resistance, we constructed isogenic *E. coli* K12 strains expressing CTX-M-15 (K12 CTX-M), CMY-2 (K12 CMY), or both enzymes (K12 CMYCTX; Fig. S1). We screened these strains against a panel of 10 different β-lactams (materials and methods) at 5 different pH values: 5, 6, 7, 8, and 9. While we saw no difference between the MIC of the wild type (K12) and the β-lactamases toward carbapenems, we saw a marked effect of pH on the MIC of these strains against certain cephalosporins (Fig. 1).

Particularly, K12 CTX-M tended toward a higher MIC at acidic pH toward cefepime, ceftazidime, and cefotaxime. In contrast, we found the opposite was the case for K12 CMY, which tended toward a higher MIC at basic pH for ceftazidime and cefotaxime, but not cefoxitin (Fig. 1A). Considering the importance of 3GC resistance, we opted to focus our attention on how ceftazidime and cefotaxime are differentially affected by CTX-M and CMY β-lactamases.

To confirm that this finding was not an artifact of our strain engineering, we tested the ceftazidime susceptibility of 13 and 14 clinical isolates expressing CMY-2 or CTX-M-15, respectively, at five different pH values (Fig. 1B). Here, we observed a similar pH/MIC relationship as previously for ceftazidime susceptibility (detailed response of each isolate can be found in Table S2).

### Temperature shows synergistic effects with pH

**Fig 2:**
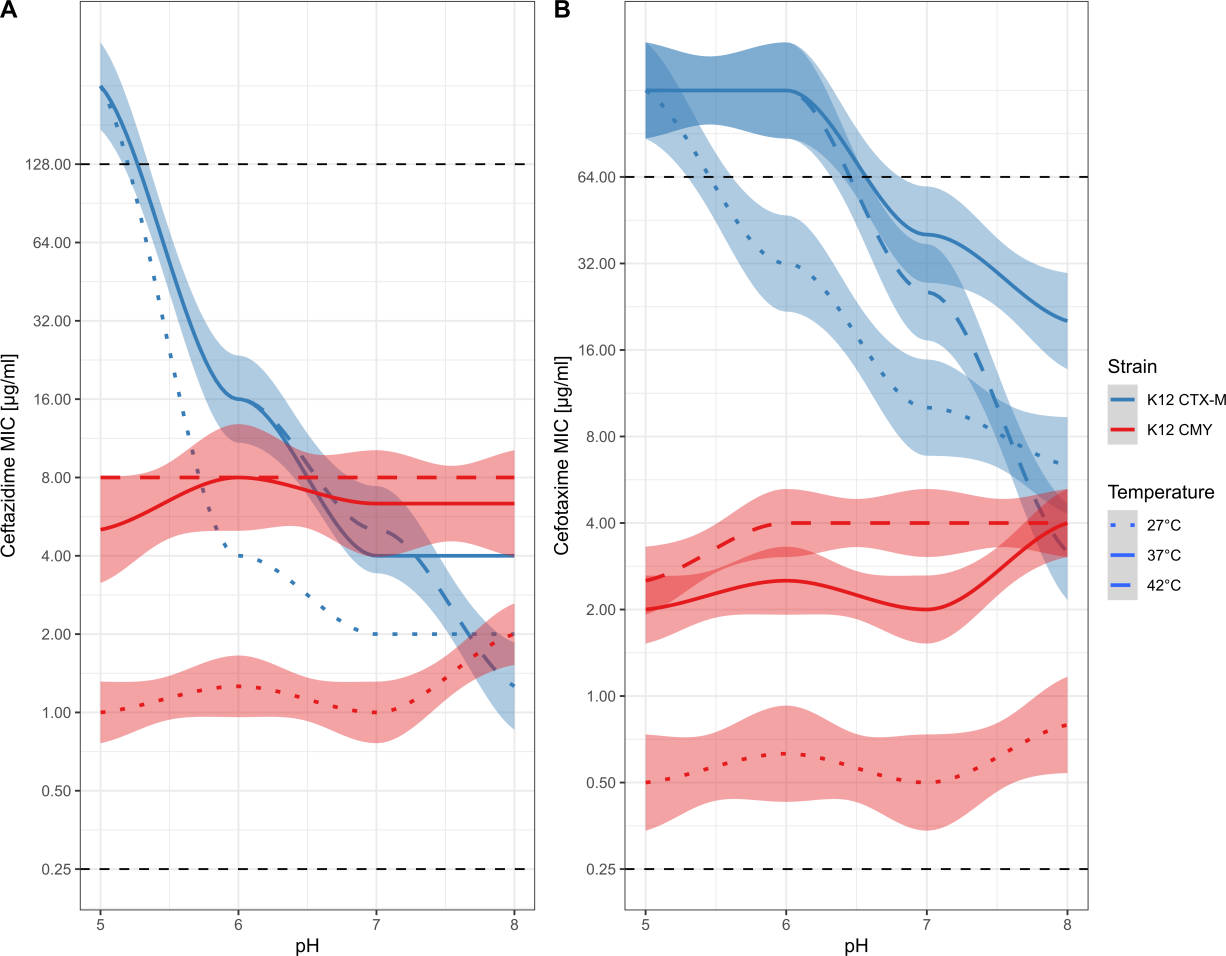
Temperature and pH variations show synergistic effects on MIC of K12 CTX-M or K12 CMY against (**A**) ceftazidime or (**B**) cefotaxime. Horizontal dashed black lines represent the maximum and minimum concentrations that were tested. The plots show the smoothed conditional means of the MIC measured for each strain at each condition (each condition was replicated three times) along with the 95% confidence interval (lighter shaded area around the lines).

MIC was determined for ceftazidime and cefotaxime to explore possible additive effects of temperatures reflecting the environment (27°C), the human host (37°C), and avian hosts (42°C). To isolate the effect of pH and temperature on each gene, we focused only on the isogenic *E. coli* K12 strains. In general, K12 CTX-M showed similar or slightly lower MIC at temperatures above 37°C, whereas K12 CMY showed increasing MICs with an optimum at 42°C, in line with its abundance in avian hosts (63) (Fig. 2). Collectively, these results suggest that pH and temperature are synergistic in their effects on MIC.

### pH and ceftazidime concentration shape the fitness landscape of CTX-M-15 and CMY-2 expressing strains

**Fig 3:**
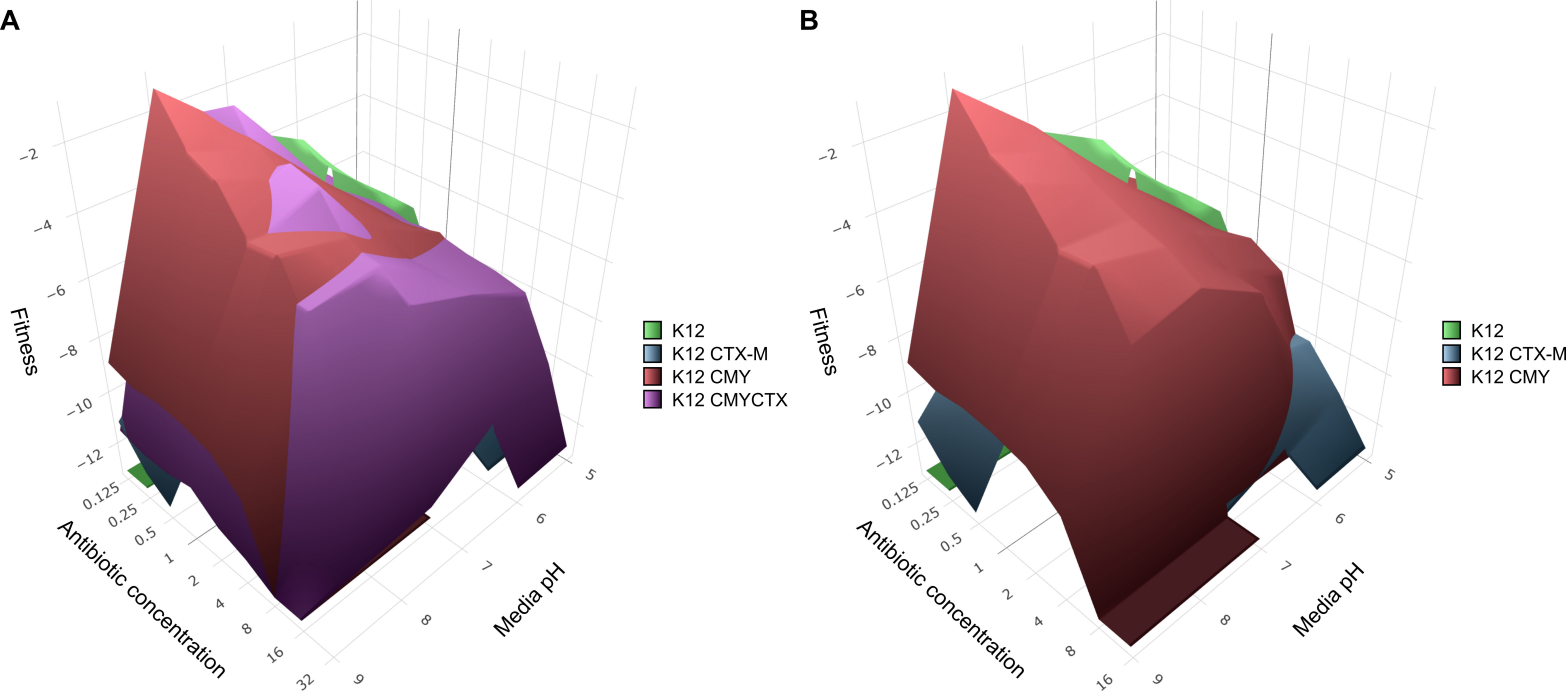
Fitness landscape of β-lactamase expressing strains, showing the most fit strain at a given ceftazidime concentration and pH. (**A**) At sub-MIC concentration (0.125–4 µg/mL) of ceftazidime, K12 CMY is fittest. On the other hand, K12 CTX-M appears to be fittest in a niche at acidic pH above 4 µg/mL ceftazidime. However, with increasing antibiotic stress, K12 CMYCTX starts to take over across all pHs assayed, illustrating how harboring multiple different β-lactamases can be beneficial to the success and survival of a strain. (**B**) Same as A but without K12 CMYCTX.

To better understand the dynamics of the β-lactamase expressing strains grown in the presence of ceftazidime at different pH conditions, we grew these strains at different ceftazidime concentrations (between 0.125 and 64 µg/mL; Fig. 3) and pH values (5, 6, 7, 8, and 9) and monitored their growth over time (OD600). From these experiments, we determined the fitness of each strain based on their growth rate and lag time (similar to reference 64, summarized in Fig. S4). Using these data, we predicted how successful each strain would be given a particular environment, as illustrated with K12 CMYCTX (Fig. 3A) and without (Fig. 3B).

At low ceftazidime concentrations and higher pH, K12 CMY appears to be the most fit strain; however, K12 CTX-M is slightly more fit around pH 6 and above 4 µg/mL ceftazidime. At high concentrations of ceftazidime (>8 µg/mL), K12 CMYCTX takes over as the most fit strain, as it is not restricted to pH-specific niches.

When we exclude the K12 CMYCTX strain from the fitness landscape, the specialization of each β-lactamase becomes much more apparent (Fig. 3B). Here, it is clearly illustrated how K12 CTX-M is most fit at low pH and K12 CMY is most fit at high pH, when the ceftazidime concentration is above 4–8 µg/mL.

**Fig 4:**
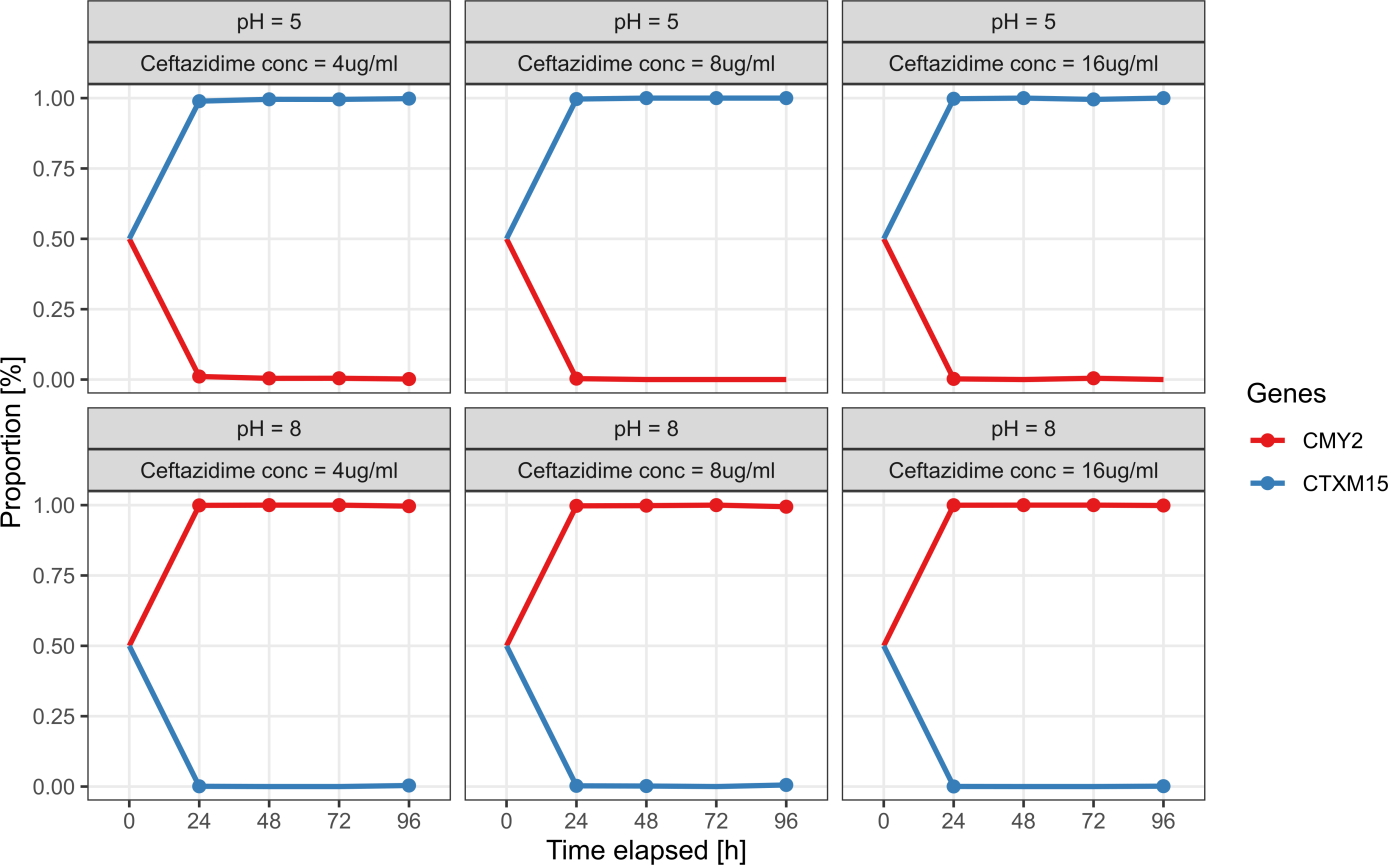
Community composition of co-cultures containing K12 CMY and K12 CTX-M. Each plot shows the proportion of strains in the co-culture over time. For each coculture, the pH and ceftazidime concentration have been indicated in panels above each plot. This co-culture was sampled and passaged into fresh media every 24 h. Solid circles indicate samples where a given strain could be detected by sequencing.

To verify these fitness predictions, we co-cultured K12 CTX-M and K12 CMY under multiple different conditions. Using long-read Oxford Nanopore sequencing (ONT), we could monitor the relative abundance of each strain in the co-cultures at multiple time points. First, we investigated the ecology of a K12 CMY + K12 CTX-M co-culture at pH 5 or 8 at three different concentrations of ceftazidime (sampled and passaged every 24 h; Fig. 4). After 24 h at each assayed condition, we observed a population shift from the initial 1:1 ratio of strains to a population consisting of just one dominant strain (>99% relative abundance). In the absence of ceftazidime, we see that K12 CMY is a more fit strain regardless of pH (Fig. S7), establishing that the addition of ceftazidime at pH five drives the selection for K12 CTX-M.

**Fig 5:**
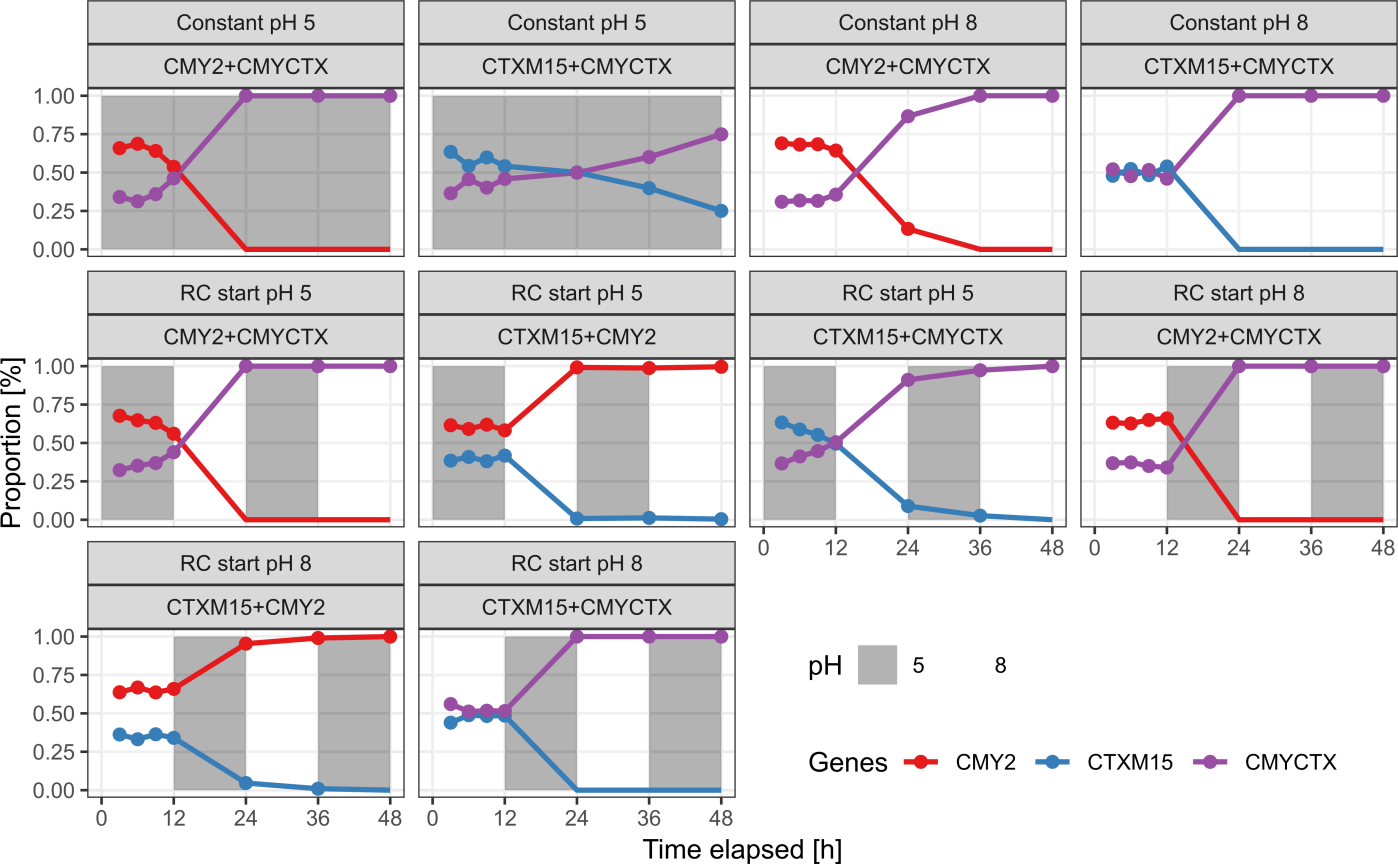
Co-cultures of strains at constant or rapidly changing (RC) pH and 4 µg/mL ceftazidime. The relative proportion of each strain has been shown in each plot, while the conditions of each co-culture have been indicated in panels above the plots. Solid circles indicate samples where the corresponding strain could be detected using sequencing. Strains below the detection limit are set to 0 proportion.

Next, we included the K12 CMYCTX strain and investigated the impact of rapidly changing the pH of the co-culture. Compared to the previous co-culture (only K12 CTX-M and K12 CMY), we used a 300× higher concentration (but still 1:1 mix) of bacteria at the beginning, and every co-culture contained 4 µg/mL of ceftazidime. This higher initial inoculum was used to allow lower-fitness strains to persist across passages under non-optimal conditions, preventing quick washout and enabling dynamic observation over time. This time, we also opted to sample more intensively during the first 12 h, and the co-culture was passaged to fresh media every 12 h. We opted not to repeat the constant pH experiments with K12 CMY and K12 CTX-M co-cultures, as we found it unlikely that this would change the result. At constant pH 5 or pH 8, we found that K12 CMYCTX was the most successful strain (using ONT sequencing), reaffirming our fitness landscape model (Fig. 4). At rapidly changing pH (between pH 5 and 8, starting at either 5 or 8 as indicated), we found that the order of fitness for the strains was K12 CMYCTX > K12 CMY > K12 CTX-M (Fig. 5). With this experimental setup, we find that in the absence of ceftazidime and, importantly, regardless of pH, K12 CMY and K12 CMYCTX are about equally fit, while K12 CTX-M is less fit than both (Fig. S8).

**Fig 6:**
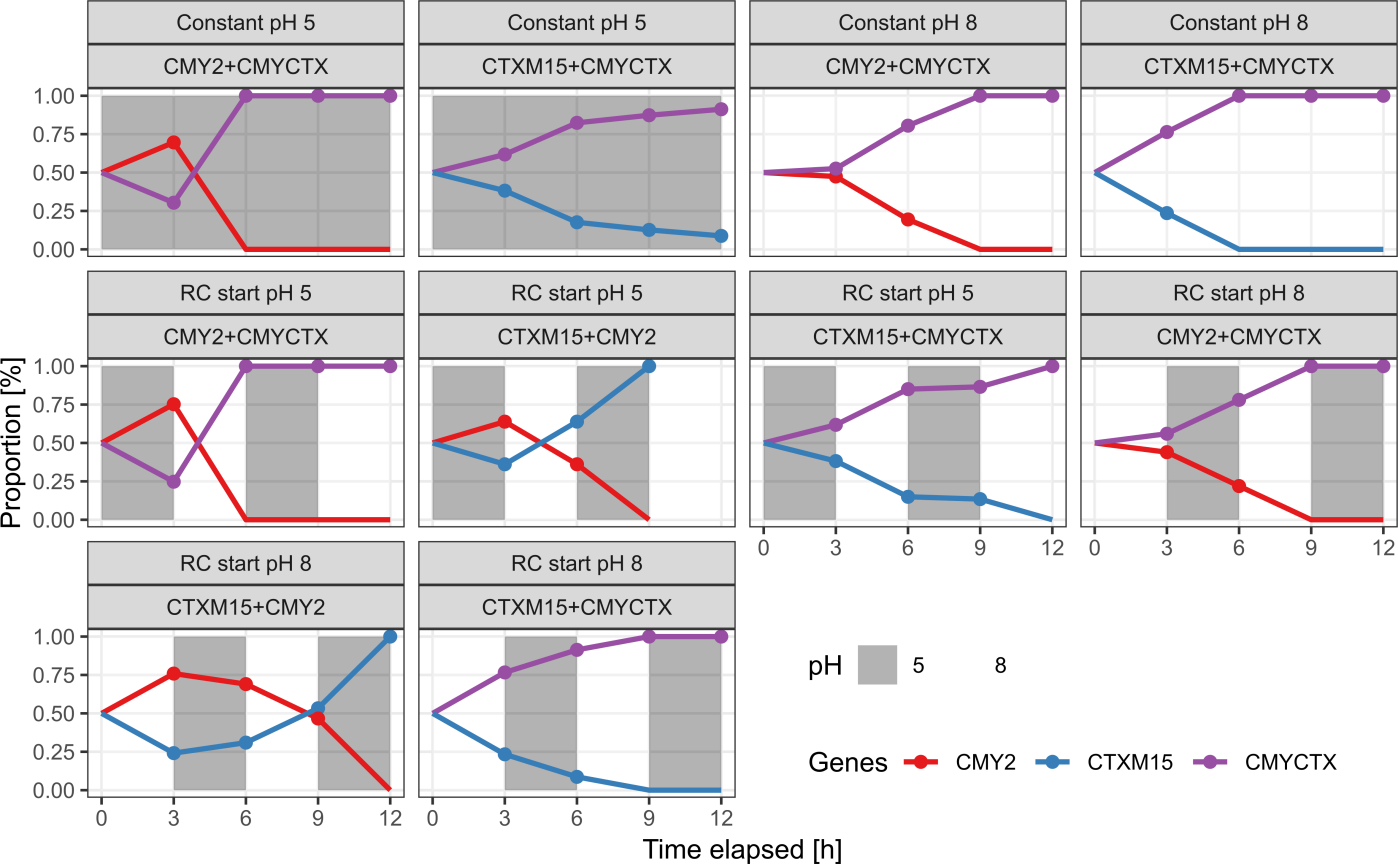
Co-cultures of strains at constant or rapidly changing (RC) pH in media supplemented with 4 µg/mL ceftazidime. The relative proportion of each strain has been shown in each plot, while the conditions of each co-culture have been indicated in panels above the plots. Solid circles indicate samples where the corresponding strain could be detected using sequencing. Strains below the detection limit are set to 0 proportion. We added an initial 0.5 proportion at *t* = 0; however, we did not sample at this time point.

Finally, we opted to investigate how the frequency of stress would affect the population of each co-culture. This time, we sampled and passaged co-cultures every 3 h for a total of 12 h. Every co-culture contained 4 µg/mL ceftazidime. Again, the K12 CMYCTX strain was dominant in every co-culture regardless of pH. Surprisingly, the K12 CTX-M strain was more successful when the frequency of pH stress was high (compared to the previous setup with lower frequency of pH changes), prevailing as the dominant strain in both rapidly changing pH co-cultures with K12 CMY (Fig. 6). It is worth noting that under rapidly changing pH, co-cultures that did not contain K12 CMYCTX quickly became “washed-out,”’ and we could not detect any DNA in the 12 h sample of the “RC Start pH 5” K12 CTX-M + K12 CMY co-culture (hence the lack of datapoints for this time). Similarly to the previous experimental setups, in the absence of ceftazidime, selection is not pH dependent (Fig. S9).

### Kinetic analysis links enzyme activity to pH-driven resistance phenotypes

To determine whether the pH-dependent resistance phenotypes were driven by changes in enzyme activity, we measured the catalytic efficiency of purified CTX-M-15 and CMY-2 β-lactamases toward nitrocefin across a range of pH values.

To measure the pH effect of both enzymes against the same substrate, we opted to use nitrocefin, as it is hydrolyzed by at least one representative from each class of β-lactamase produced by bacteria (65). We found that the kinetics of both β-lactamases closely matched the pH-dependent pattern that we observed in previous experiments. CTX-M-15 displayed the highest turnover (Kcat) at acidic to neutral pH, with an optimum at pH 6 (Table 1), while CMY-2 had an optimum at pH 8 to nitrocefin.

While it would have been highly relevant to compare the activity of both enzymes to ceftazidime across the same pH range, we were unable to measure the CMY-2 hydrolysis rates, consistent with previous reports of low catalytic *in vitro* efficiency for this enzyme (66, 67) (Kcat ≤ 0.01 s⁻¹, Km ~ 0.02 μM at pH 7). We did, however, measure the ceftazidime hydrolysis rates for CTX-M-15 toward ceftazidime, which matched the results found for nitrocefin hydrolysis (activity was inversely correlated with pH; Fig. S6).

**TABLE 1:** Enzyme kinetics of β-lactamases to nitrocefin at different pHs. Kinetic parameters were determined using non-linear least squares fit to the Michaelis Menten equation

Assay pH	CTX-M-15			CMY-2		
	Km (µM)	Kcat (s^−1^)	Kcat/Km (s^−1^µM^−1^)	Km (µM)	Kcat (s^−1^)	Kcat/Km (s^−1^µM^−1^)
5	8.63	192.55	22.32	34.32	255.60	7.45
6	12.56	195.55	15.57	2.79	160.96	57.73
7	6.46	164.62	25.47	31.39	261.63	8.33
8	10.61	68.84	6.49	177.21	686.54	3.87
9	25.85	39.91	1.54	69.24	458.31	6.62

### *In silico* molecular modeling shows that pH alters active site electrostatics, with limited impact on substrate binding

**Fig 7:**
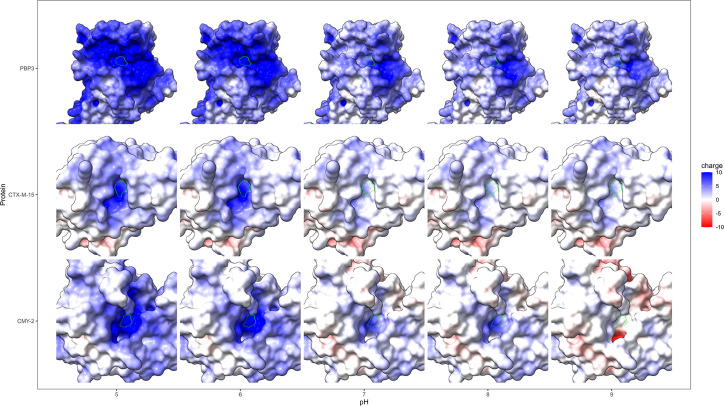
Influence of pH on electrostatic surface potentials of β-lactamases. Electrostatic potentials were calculated for proteins PBP3 (pdb: 7onw), CTX-M-15 (4hbt), and CMY-2 (1zc2) and used to color the surface. For each protein, we focused on the active site groove, centered around the catalytic serine (S70 for CTX, S64 for CMY, and S307 for PBP3, marked with a bright green ring).

To determine why pH seems to affect the two β-lactamases differentially, we utilized molecular dynamics and docking to investigate how it affects the proteins and to support our mechanistic interpretation of the experimental findings. First, we modeled the surface charge of each peptide at different pH conditions. We found that at acidic pH (5, 6), the active site of each peptide is positively charged (Fig. 7), which is a known phenomenon (oxyanion hole) (68, 69) as it is thought to stabilize the negative charge of a negatively charged oxygen (for instance, the catalytic serine in both β-lactamases). At pH 7, however, there is a sudden shift in the surface charge of CTX-M-15 to neutral near the active site, while CMY-2 remains positively charged. CMY-2 appears to have a more gradual change in active-site surface charge from positive to neutral, as a response to increased pH. These differences in local surface charge near the active site may account for some of the contrasting effects that pH has on the MIC of strains expressing these β-lactamases.

Focusing on the substrates ceftazidime and cefotaxime, they are primarily negatively charged between pH 5–9, where cefotaxime has a net charge of −1 across the entire pH interval (70). The net charge of ceftazidime, on the other hand, decreases with increasing pH from −0.66 at pH 5 to −1.96 at pH 9 (71). The importance of negative electrostatic potential in β-lactam substrates has previously been found important for their bactericidal activity (72). Following this logic, a similar opposite-signed electrostatic potential may be important for the activity of β-lactamases.

Using AutoDock Vina, we then investigated the binding energies of ceftazidime, cefotaxime, and nitrocefin to the active sites of CTX-M-15 and CMY-2 at different pH conditions. Similarly, we were also interested in how close the catalytic serine of each β-lactamase was positioned to the oxygen in the β-lactam moiety of each substrate, as the first step of hydrolysis involves acylation of the catalytic serine with this oxygen (73).

**Fig 8:**
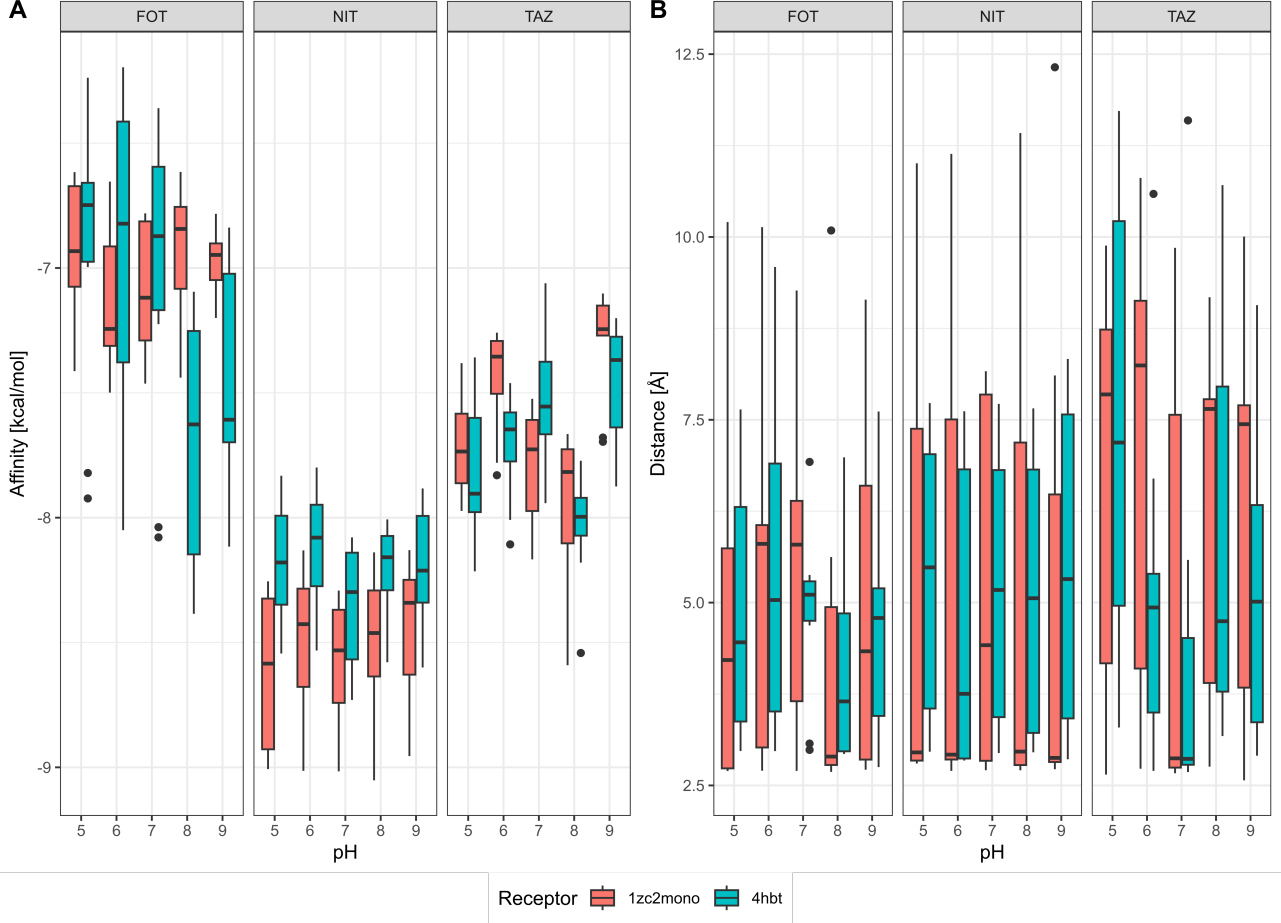
Molecular docking of β-lactam substrates to β-lactamases at different pHs. (**A**) The affinity of different substrates (top of each plot denotes ligand; abbreviations: FOT, cefotaxime; NIT, nitrocefin; TAZ, ceftazidime) to β-lactamases CMY-2 (1zc2) and CTX-M-15 (4hbt). (**B**) The distance between the catalytic serine of each β-lactamase and the β-lactam oxygen.

Focusing on the affinity of β-lactamases to different substrates at different pH conditions (Fig. 8A), we found that there are only minor differences caused by pH (in the magnitude of ±1 kcal/mol) and that, for the most part, there are no substantial differences in binding energies when comparing the two β-lactamases. Similarly, the distances between the catalytic serine and the β-lactam show even higher similarity across pH and β-lactamase. Both of these results indicate that the mechanistic background for pH-mediated differences is perhaps not found within the active site of the β-lactamase but may be related to protein folding, stability, or even the position of nucleophilic water within the active site (73), which cannot be determined with these methods.

## DISCUSSION

In this study, we investigated how pH conditions, such as those found in different hosts, infection sites, tissues, and cell compartments, can influence ARGs and what implications this has for AST. By expressing different β-lactamases in isogenic strains of *E. coli*, we compared their specific activity under different conditions. We found that each β-lactamase was associated with contrasting pH optima: CTX-M-15 with acidic and CMY-2 with alkaline conditions. Strikingly, CTX-M-15 goes from resistant at pH 7 to susceptible at pH 9, indicating that it might be possible to treat resistant infections by modifying the pH of the infection site. On the other hand, using electron microscopy (Supplementary Information: Fig. S2), we found that ceftazidime treatment of the same strain at pH 5 may induce a potentially immune-evading (74) filamentous phenotype in *E. coli* K12, which is a recipe for recurrent infection (however, we did not observe the same in the clinical isolates expressing CTX-M-15, suggesting this may be a consequence of our strain construction or a feature of the K12 genotype). *Salmonella* has been found to produce an alternative penicillin-binding protein under acidic conditions (such as the phagosome), which is less susceptible to ceftriaxone. On the other hand, methicillin-resistant *Staphylococcus aureus* becomes susceptible in the acidic environment of the phagolysosome due to pH (24, 75).

In rapidly changing pH environments (Fig. 5 and 6), we observed a reversal in the relative fitness of the strains: CTX-M-15-expressing strains outcompeted CMY-2 strains even under conditions where CMY-2 had higher enzyme activity at static pH. Examples of rapidly changing environments include bacterial transit along the gut, vaginal colonization from the gut, soil acidification after rainfall, factory acid waste water, endophytic colonization of certain plants, and phagocytosis (including location in the phagosome) (24, 29, 71, 75–81).

This suggests that enzyme kinetics alone cannot fully explain the observed fitness patterns, as they do not predict the success of CTX-M-15 over CMY-2 in the rapidly changing pH environment. It is possible that pH shifts influence β-lactamase-expressing strains differentially due to bacterial stress responses, differences in expression cost, enzyme stability, potential toxicity of enzymes, or periplasmic maintenance of enzymes. Considering the result of all the co-cultures, we speculate that CMY-2 may be rapidly denatured at pH 5, rendering K12 CMY susceptible to ceftazidime as opposed to K12 CTX-M in the short term (RC Start pH 5 and RC Start pH 8 containing K12 CTX-M and K12 CMY; Fig. 6). However, given enough time, the acid shock response(s) (82, 83) would “kick in” and reduce this fitness burden as seen for the 12 h interval co-culture where K12 CMY takes over both RC Start co-cultures (RC Start pH 5 and RC Start pH 8 containing K12 CTX-M and K12 CMY; Fig. 5). On the other hand, a large part of the CTX-M-15 pool is being exported outside of the cell (84), where it may be (i) more stable to pH shifts and (ii) unable to sequester the pool of periplasmic chaperones, responsible for preventing the aggregation of acid-unfolded proteins and assisting with refolding in the acid-recovery phase (85). Previous studies have shown that even a small fraction (0.1%) of misfolded total cellular proteins can incur a fitness cost in yeast (86). Another factor to consider is that the bactericidal effect of β-lactams is time dependent (87), enabling the success of the K12 CTX-M at its MIC for 3 but not 12 h.

Seemingly, CMY-2 is a much more successful β-lactamase in poultry (63) than in humans, which may be explained by the pH and temperature differences between poultry and human. The pH of the GI tract in chicken is mostly stable close to neutral pH (88, 89) (after the gizzard), which would favor acquisition and maintenance of CMY-2 as opposed to the more human-favoring CTX-M-15 (63, 90–93), which may withstand the comparably more variable but mostly acidic human GI tract (30–33). Interestingly, the activity of CMY-2 seemed to increase with temperature and would especially at 42°C more likely to outcompete the CTX-M-15 positive strain. The implications of this are that not only does the accumulation of multiple different β-lactamases increase the fitness of an isolate to a wider range of substrates (60), but it may also increase its fitness to similar substrates across a wider range of environments, making the benefit twofold.

We posit that the reason why the pH-dependent effects on MIC and enzymatic turnover rates largely match is because both genes are expressed at similar rates (94), and second, because β-lactamases are for the most part either exported outside the cell, in the case of CTX-M-15, or to the periplasm where there is no pH homeostasis in *E. coli* (84, 95–97). Both CTX-M-15 and CMY-2 have predicted signal peptides (98), which use the Sec/SP1 pathway to direct their transport to the periplasm. This leaves β-lactamases highly exposed to extracellular pH, and as such, we propose that the results of this study may be broadly applicable to other β-lactamases and other pathogens that excrete them. On the other hand, very few microorganisms besides *E. coli* have had their periplasmic pH probed (to the knowledge of the authors); however, *Helicobacter pylori* is known to maintain periplasmic pH near pH 6 (96). Furthermore, bacteria are known to employ a myriad of protective mechanisms against pH stress (83) and other environmental stresses (redox, temperature, etc), which must be studied further in human pathogens to properly guide healthcare and policy makers.

While the acquisition of multiple ARGs is often attributed to plasmid-mediated horizontal gene transfer, it remains unclear why selective pressure does not purge less effective or redundant genes. Our results suggest that ARG redundancy here for β-lactamases may provide a bet-hedging advantage, where maintaining multiple resistance genes ensures survival across fluctuating environmental conditions such as pH shifts encountered in diverse host niches. Inconsistent pH profiles, for example, between gut, bladder, bloodstream, and inflamed tissues, may favor strains that carry a broader arsenal of enzymes with different pH optima, ensuring resistance across a wider range of conditions. This could also contribute to the success and persistence of certain epidemic clones of *E. coli* that co-harbor both CTX-M and CMY enzymes.

Our findings further highlight possible limitations of current AST, which is typically performed under fixed laboratory conditions (pH 7.2–7.4). Our data show that both enzyme activity and strain fitness vary dramatically across the physiological pH range found in host environments. Consequently, MIC values obtained under standard AST may poorly predict treatment outcomes in infections occurring at low or high pH (e.g., in urine, abscesses, or GI sites). Incorporating environmental factors such as pH into AST or clinical decision-making could improve the accuracy of resistance phenotyping and enable tailored interventions, including pH modification therapies where applicable.

## MATERIALS AND METHODS

### Bacterial strains, plasmids, and culture conditions

Bacterial strains and plasmids used in this study have been listed and described in Table 2. Cultures of *E. coli* were routinely cultured in LB at 37°C in a shaking incubator at 200 rpm, unless otherwise stated. Cultures were stored at −80°C by the addition of glycerol to a final concentration of 20% glycerol. For maintenance or selection of plasmids, gentamicin or ampicillin was supplemented at 15 µg/mL or 100 µg/mL, respectively. Electrocompetent *E. coli* cells were prepared and electroporated according to the Biorad MicroPulser protocol (99) using 1 mM cuvettes. A total of 27 clinical isolates were chosen on the basis of them being *E. coli*, either carrying CTX-M-15 or CMY-2, and harboring no other resistance mechanisms to β-lactam class compounds (as evaluated using WGS data and AMRFinderPlus [100] [v4.0.19] with parameter—organism Escherichia).

**TABLE 2:** Strains and plasmids used in this study^*a*^

Strains		
Name	Comment	Source
*Escherichia coli* DH5αpir	Plasmid maintenance	Lars Jelsbak
*Escherichia coli K12 MG1655*	Laboratory strain	Reference 101
MG1655-sfGFP-blaCTX-M-15	Source of blaCTX-M-15	Reference 102
MG1655-sfGFP-blaCMY-2	Source of blaCMY-2	Reference 102
K12-CTX	K12 attTn7::pMA1 CTX-M-15	This study
K12-CMY	K12 attTn7::pMA1 CMY-2	This study
K12-CMYCTX	K12 attTn7::pMA1 CMYCTX	This study
TWIW_02_DEU_MAG_BI_061	CMY-2 expressing clinical isolate	Reference 103
TWIW_02_DEN_HVI_BM_056	CMY-2 expressing clinical isolate	Reference 103
TWIW_02_CHE_ZUR_BX_012	CMY-2 expressing clinical isolate	Reference 103
2010_60_1061_1	CMY-2 expressing isolate	Reference 104
2010_60_5498_4	CMY-2 expressing isolate	Reference 104
2010_60_5499_19	CMY-2 expressing isolate	Reference 104
2010_60_7075_19	CMY-2 expressing isolate	Reference 104
2010_60_7077_57	CMY-2 expressing isolate	Reference 104
2010_60_7077_7	CMY-2 expressing isolate	Reference 104
2028_7	CMY-2 expressing isolate	Reference 104
2028_8	CMY-2 expressing isolate	Reference 104
2067_2	CMY-2 expressing isolate	Reference 104
2115_5	CMY-2 expressing isolate	Reference 104
TWIW_01_GHA_SEK_008	CTX-M-15 expressing clinical isolate	Reference 103
TWIW_02_NGA_ILE_BN_040	CTX-M-15 expressing clinical isolate	Reference 103
TWIW_02_ALB_TIR_AI_026	CTX-M-15 expressing clinical isolate	Reference 103
TWIW_02_KAZ_ALM_CD_036	CTX-M-15 expressing clinical isolate	Reference 103
TWIW_02_LTU_KAU_BG_025	CTX-M-15 expressing clinical isolate	Reference 103
TWIW_01_PAK_PES_036	CTX-M-15 expressing clinical isolate	Reference 103
TWIW_02_NGA_ABU_AD_008	CTX-M-15 expressing clinical isolate	Reference 103
TWIW_02_PAK_PES_AA_062	CTX-M-15 expressing clinical isolate	Reference 103
TWIW_01_CZE_PRA_051	CTX-M-15 expressing clinical isolate	Reference 103
TWIW_02_PAK_PES_AA_013	CTX-M-15 expressing clinical isolate	Reference 103
TWIW_02_CHE_ZUR_BX_047	CTX-M-15 expressing clinical isolate	Reference 103
TWIW_01_NGA_ABU_020A	CTX-M-15 expressing clinical isolate	Reference 103
TWIW_02_FRA_LIL_AK_033	CTX-M-15 expressing clinical isolate	Reference 103
TWIW_01_TUR_ORT_046	CTX-M-15 expressing clinical isolate	Reference 103
Plasmids		
pUC18R6k-mini-tn7T-gm	Tn7 plasmid	Addgene plasmid #65,022 (105)
Tn7 helper plasmid	Tn7 helper plasmid	Addgene plasmid #141,161 (106)
pUC57 BG28 BCD2	Synthesized DNA in plasmids	This study
pMA1 BG28	pUC18R6k-mini-tn7T-gm with regulon BG28 BCD2 integrated	This study
pMA1 CTX-M-15	pMA1 BG28 with CTX-M-15 integrated at NcoI site	This study
pMA1 CMY-2	pMA1 BG28 with CMY-2 integrated at NcoI site	This study
pMA1 CMYCTX	pMA1 BG28 with CMYCTX integrated at NcoI site	This study

^*a*^ TWIW, two weeks in the world.

### Media

CAMHB was prepared according to the manufacturer’s specifications. pH-adjusted CAMHB (pHCAMBH) was prepared by the addition of HCl (4 M) or NaOH (5 M) to pHs 4.95, 5.8, 6.8, 7.95, and 9.15 and autoclaved. Post-autoclaving, we found that the broth pH changed slightly, which is why we adjusted the broth to be slightly more acidic pre-autoclaving. One exception was pH 9, which had to be adjusted to 9.15 pre-autoclaving to reach the target pH. The pH of autoclaved (room temperature) PHCAMBH was verified to be within ±0.1 of the target pHs (5, 6, 7, 8, and 9) using a pH probe. SOC medium(20 g/L tryptone, 5 g/L yeast extract, 10 mM NaCl, 2.5 mM KCl, 20 mM MgSO_4_ autoclaved, and supplemented with glucose to 20 mM with sterile-filtered glucose) was used to rescue electro-transformants. Ceftazidime stock solution was prepared from powder at 4.5 mg/mL in PBS pH 7, aliquoted, and frozen at −20°C. Nitrocefin stock solution was prepared from powder at 20 mg/mL in DMSO and diluted 20× in PBS; this was aliquoted and frozen at −20°C. 10× Universal buffer 4 (107) (200 mM Sodium acetate, 200 mM MES, and 200 mM HEPES) was prepared, filter sterilized (0.2 µm), and adjusted to either pH 4 or 9 with HCl and NaOH, respectively. These were mixed in empirically determined ratios to prepare buffers at specific pHs (in [vol/vol]: pH 5: 76.6% pH 4, 23.4% pH 9; pH 6: 54.4% pH 4, 45.6% pH 9; pH 7: 30.6% pH 4, 69.4% pH 9; pH 8: 6.8% pH 4, 93.2% pH 9; pH 9: 100% pH 9).

### Plasmid construction: constitutively expressed single and double-gene constructs

For this study, we chose the mini-Tn7 transposon that is proficient in single-copy integration of DNA constructs at a high rate at a single site on the chromosome of a plethora of bacterial species. The pUC18R6k-mini-tn7T-Gm plasmid was then prepared for cloning by PCR amplification, using primers that introduce a NotI and NcoI to each end of the linearized plasmid. We then digested and ligated synthesized oligonucleotides (BG28 [agt**gcggccgc**a*CTAGGTTGACATGGATATAATGTATGTA*GGG], BCD2 [CCCAAGTTCACTTAAAAAGGAGATCAACAATGAAAGCAATTTTCGTACTGAAACATCTTAATCATGCTAAGGAGGTTTTCTAATGatcagattccaggcggtg**ccatgg**tatt]; restriction sites for NotI and NcoI are shown in bold [in that order], and promoter sequence and translation initiation elements are in italics and underlined, respectively, and in capital letters) together with the mini-Tn7 plasmid to create a vector pMA1. This vector is a platform, where we can integrate a gene-of-interest (GOI) by Gibson assembly that is transcribed and translated in a predictable and controlled manner. We opted to use a weakly expressed constitutive promoter from a synthetic promoter library BG28 (108) as well as a bicistronic translation initiation element, BCD2(94), which reduces GOI-specific effects on translation, to enable a much better comparison of different β-lactamases, independent of their sequence.

pMA1 was then linearized by PCR, which prepares it for Gibson assembly with a GOI (also PCR amplified to add homology tails). Gibson assembly was then performed according to the manufacturer’s instructions (New England Biolabs, Gibson Assembly Master Mix) with the exception that it was incubated at 50°C for 1 h. The assembly mix was then diluted in Milli-Q H_2_O or cleaned up (Monarch PCR and DNA Cleanup kit) prior to electroporation into electro-competent DH5αpir cells. Correct plasmid assembly was selected for by the addition of gentamicin. We verified the correct integration of GOIs into this plasmid using PCR and Sanger sequencing.

To study the effect of expressing multiple β-lactamases, we prepared constructs that express two genes. This was accomplished by PCR amplifying pMA1 single-gene constructs to add flanking NcoI restriction sites to the GOI. Using FD NcoI (Thermofisher), we linearized a pMA1 GOI-1 plasmid and ligated it to the NcoI-digested PCR-amplified GOI-2 to create a two-gene expression vector.

### Tn7 integration of constructs

We opted to test the constructs using the *E. coli* K12 MG1655 strain. To prepare MG1655 for tn7 tagging, it was made electrocompetent according to Biorad protocol, transformed using 1 µL of purified Tn7 helper plasmid, and plated on LBA + Amp100 plates grown at 30°C. To induce Tn7 transposon expression, a culture of this strain was grown at 30°C until OD 0.25, where L-arabinose was added to a final concentration of 0.2%. The cells were grown an additional 40 min (to OD = 0.4–0.6) and incubated on ice. Cells were then made electrocompetent, and 40 µL of the resulting cells were used for each transformation. Cells were rescued by the addition of 1 mL SOC medium and grown at 37°C for 1 h prior to plating them on LBA + Gm15 plates.

### AST and growth kinetics

Cultures were adjusted to 0.5 McFarland and then diluted 300× in Mueller-Hinton broth prior to being tested for antimicrobial susceptibility. Cultures were assayed using commercial MIC panels to test multiple substrates at once or in defined microtiter MIC assays that were prepared prior to each experiment. Using the commercial MIC panel: EUVSEC2 (Thermo Scientific), we deposited 50 µL culture into each well to test 10 different individual substrates and substrate combinations (substrates: cefoxitin, ertapenem, imipenem, meropenem, ceftazidime, cefepime, cefotaxime, and temocillin). Ceftazidime and cefotaxime were additionally tested in combination with 4 µg/mL clavulanic acid. These were grown for 20–22 h in a 37°C incubator. While we tested QC strain *Acinetobacter baumannii* 2012-70-100-69 under each condition (pH, temperature), no QC ranges exist for non-standard conditions.

To determine the growth kinetics of strains at different pH levels and concentrations of ceftazidime, we prepared broth microdilution assays and monitored the growth of strains in each well of a 96-well microtiter plate using a plate reader (ThermoFisher Multiskan SkyHigh). Growth was monitored for 22 h at 37°C, and wells were read at 600 nm every 4 min with “medium” shaking between reads. Each condition was assayed in triplicate. From this data, we estimated growth rates and lag time from each condition using linear regressions of log2-transformed optical density values. Lag time was estimated according to equation 1.

Equation 1: estimation of lag time from kinetic growth, where min(logOD) is the lowest recorded log2 transformed OD value for a given condition, *I* is the intercept of the linear regression used to estimate growth rates, and *r* is the slope. This equation is based on the formula for a line, solving for *x* in *y* = *ax* + *b*. This equation is used to calculate the time that the linear regression intersects with the lowest measured OD of a given kinetic growth curve.


$$l=\frac{min\left(logOD\right)-I}{r}.$$

Using the growth kinetics, we simulated growth of each strain under each condition to create a fitness landscape of strains, incorporating mean lag times and mean exponential growth rates according to equation 2. As we calculated slopes (rates) using log2 transformed OD data, we transformed these to exponential growth rates by calculating 2 to the power of the slope $m=2^{r}$, which could be used in equation 2. For clarity, we have illustrated the result of using these equations (Fig. S10).

Equation 2: simulating exponential growth of cells where *c* is the number of cells after growth, *c*0 is the initial number of cells, *m* is the exponential growth rate, *t* is the time of growth, and *l* is the lag time. This equation is based on the formula for exponential growth.


$$c=c_{0}*{\left(m\right)}^{t-l},\;\;t>l.$$

Finally, we determined the fitness according to equation 3.

Equation 3: fitness of strains is defined as the log2 fold change of cells to the number of starting cells, c0.


$$\text{Fitness}=\text{log2}\left(\frac{c}{c_{0}}\right).$$

### Co-culturing experiment at constant or changing pH under β-lactam pressure

Single colonies were used to inoculate 5 mL Mueller Hinton, which was grown overnight at 37°C, 200 rpm for up to 20 h. A total of 0.5 McFarland cultures were prepared from overnight cultures, and strains were mixed 1:1 in bioreactors containing pH adjusted media (pH 5 or 8) supplemented with ceftazidime to 4, 8, or 16 µg/mL. Co-cultures were incubated at 37°C, 200 rpm for up to 24 h, depending on sampling and passaging scheme. Co-cultures were sampled during or after growth by aseptically transferring 2 mL to an Eppendorf tube, which was pelleted by centrifugation (18 kg, 1 min) and frozen after discarding the supernatant (samples were frozen at −80°C for short-term storage prior to DNA extraction). After a full growth period (up to 24 h), co-cultures were passaged to fresh media by transferring 1 mL spent culture into 19 mL fresh media (supplemented with ceftazidime) and re-incubated. Each co-culture was passaged to fresh media three times for each experimental setup, totaling four growth periods under different conditions. We passaged the co-cultures so we could investigate how changes in pH (between 5 and 8 or vice versa) influenced the fitness of strains.

### Protein expression and purification

CTX-M-15 and CMY-2 genes were synthesized with a C-terminal HIS tag (6 His residues), cloned into vector pET30a, and transformed into *E. coli* BL21 Star (DE3) for protein expression. BL21 was grown in TB medium supplemented with kanamycin and incubated at 37°C at 200 rpm until OD600 = 1.2. The cultures were then induced by the addition of IPTG and grown at 15°C for 16 h. Cells were harvested by centrifugation, and pellets were resuspended in lysis buffer followed by sonication. Purified proteins were obtained by two-step purification using Ni column + Chromdex 200 column and sterilized by 0.22 µm filter. Proteins were quantified with Bradford protein assay using BSA as standard, while purity was determined by SDS-PAGE.

### Enzyme kinetics

To determine enzyme kinetics at different pH levels, we opted to use the “universal buffer 4” (UB4), as it allows the decoupling of buffer-induced changes from pH and temperature changes (107): supplemented with 100 µg/mL bovine serum albumin at pHs 5, 6, 7, 8, and 9 and at least two different substrate concentrations in a Corning UV 96-well microtiter plate. Using the ThermoFisher Multiskan SkyHigh plate reader, we monitored the absorbance of 100 µL samples over time every 10 s at 37°C to determine the kinetic parameters of recombinant CTX-M-15 or CMY-2 for nitrocefin (at 390 and 490 nm). For nitrocefin, rates were determined at initial concentrations of 100, 10, or 6.5 µg/mL.

Non-linear least-squares was used to fit data to the Michaelis-Menten equation with R package renz to estimate Km and Kcat for each enzyme/substrate/pH combination using at least three replicates (for both starting concentrations). Outliers were detected using ggplot2 boxplot and removed.

### Molecular modeling

ChimeraX 1.9 was used to prepare a monomeric 1zc2 structure and for visualization of proteins and protein charges. Surface charge of proteins CTX-M-15 and CMY-2 (pdb: 4hbt and 1zc2, respectively) was determined using pdb2pqr to estimate titration states at different pHs (v3.6.1 with parameters --ff PARSE --titration-state-method=propka --drop-water and --with=ph from 5 to 9), and electrostatics were solved with APBS (v.3.4.1).

Proteins with estimated titration states (from pdb2pqr) were also used for molecular docking and were prepared with MGLtools (v1.5.7 prepare_receptor4.py with parameters -C -U nphs_lps -v). Ligands were prepared with molscrub (v0.1.1) and Meeko (v0.6.1), using the SMILES notations for ceftazidime, cefotaxime, and nitrocefin at pHs 5–9 (with scrub.py parameters –skip_tautomers --ph_low = --ph_high). Docking was carried out with Autodock Vina (v 1.2.6) with parameters --exhaustiveness = 32, energy_range = 4 in a 30 × 30 × 30 Å box around the active site of either 1zc2 (center_x = −11.041, center_y = 45.568, and center_z = 27.517) or 4hbt (center_x = −6.018, center_y = 6.991, and center_z = 17.060).

### DNA purification sequencing and bioinformatics

DNA from bacterial suspensions or pellets was purified using the Quick-DNA Fungal/Bacterial Miniprep Kit (Zymo) and the Quick-DNA HMW MagBead kit (Zymo) similar to reference 109. DNA concentrations were quantified using a Qubit 4 Fluorometer (Invitrogen, Cat. No. Q33238), and samples were kept at 4°C prior to library preparation. For each sample, 1 μg of DNA was used to prepare sequencing libraries with the Ligation gDNA Native Barcoding Kit 24 V14 (SQK-NBD114.24, Oxford Nanopore Technologies, Oxford), following the manufacturer’s protocol with minor adjustments similar to reference 109. Libraries were subjected to sequencing on PromethIon (Oxford Nanopore Technologies) with FLO-PRO114M flowcells and basecalled using GuppyBasecaller (v7.2.13) with super-accurate basecalling option.

To create a high-quality reference K12 genome for strain comparison, we additionally sequenced the *E. coli* K12 MG1655 DNA on the NextSeq 500. Short reads were processed using fastp (v0.23.4) with flag “-c,” and long reads from PromethIon (as described above) were filtered with Filtlong (v0.2.1) with flag “--target_bases 250,000,000” for approximate 50× depth. Both short and long reads from the K12 MG1655 strain were utilized for *de novo* hybrid assembly using Unicycler (v0.5.1). K12 CMY and K12 CTX genomes were sequenced and assembled with Unicycler as described above using only short reads. The K12 CMYCTX genome was sequenced on PromethIon and assembled with Flye. Using CLC Main Workbench (v22.0.2), we blasted genomes to references to verify the integrity of chromosomal inserts.

To quantify abundances of strains present in the co-culture samples, we created a database of β-lactamase genes (and combinations) using KMA (v1.4.15) with parameters (-k 28). We mapped reads from co-cultures to this database with KMA parameters (−1t1 -ID 99 -gapextend 2 -penalty 3 - gapopen 5 -a -ont). The relative abundances were converted to proportions using an R script.

### Data analysis

Data and analyses were performed using Rstudio (2024.04.0 build 735) scripts with packages readr, ggplot2, dplyr, renz, foreach, doParallel, lubridate, ggpubr, cowplot, magick, plotly, and Rcolorbrewer.

## Data Availability

All sequencing data for this study have been published in public databases under bioproject PRJEB94045. Raw data and scripts for analysis are publicly available at https://github.com/MAphd/PHMIC

## SUPPLEMENTAL MATERIAL

Alternate 3D model from Fig. 3 aem.01775-25-s0001.html 3D model from Fig. 3, but without the CMYCTX construct.

3D model from Fig. 3 aem.01775-25-s0002.html 3D model of the fitness landscape of the different isogenic strains containing CTX and CMY β-lactamases.

Sheet S1 aem.01775-25-s0003.pdf Overview of the sequencing data generated as part of the project.

Supplemental material aem.01775-25-s0004.pdf Figures S1 to S10; Tables S1 and S2.

Supplemental legends aem.01775-25-s0005.pdf Legends supplemental HTML files.

ASM does not own the copyrights to Supplemental Material that may be linked to, or accessed through, an article. The authors have granted ASM a non-exclusive, world-wide license to publish the Supplemental Material files. Please contact the corresponding author directly for reuse.
