# Supplementary material for: Contrasting pH optima of β-lactamases CTX-M and CMY influence Escherichia coli fitness and resistance ecology
Source: Appl Environ Microbiol. 2025 Dec 29;92(1):e01775-25. doi: 10.1128/aem.01775-25 (PMC12863049; doi:10.1128/aem.01775-25)
Supplement: Sheet S1 — Overview of the sequencing data generated as part of the project. [file aem.01775-25-s0003.pdf]

Supplementary sheet 1: metadata-sheet linking sequencing data to samples, including accession numbers

Sample	ACCESSION	Type	Purpose	Description
DTU_2024_1036870_1_SI_MA068	ERS25269316	Single isolate	Strain validation	K12 CMY strain
DTU_2024_1036872_1_SI_MA080	ERS25269317	Single isolate	Strain validation	K12 CTX-M strain
DTU_2024_1036962_100_MG_2_5_4	ERS25269318	Metagenomic	Co-culture	Co-culture sample at day 2, pH 5, 4μg/ml (Figure 4)
DTU_2024_1036963_100_MG_3_5_4	ERS25269319	Metagenomic	Co-culture	Co-culture sample at day 3, pH 5, 4μg/ml (Figure 4)
DTU_2024_1036964_100_MG_4_5_4	ERS25269320	Metagenomic	Co-culture	Co-culture sample at day 4, pH 5, 4μg/ml (Figure 4)
DTU_2024_1036965_100_MG_5_5_4	ERS25269321	Metagenomic	Co-culture	Co-culture sample at day 5, pH 5, 4μg/ml (Figure 4)
DTU_2024_1036966_100_MG_2_5_8	ERS25269322	Metagenomic	Co-culture	Co-culture sample at day 2, pH 5, 8μg/ml (Figure 4)
DTU_2024_1036967_100_MG_3_5_8	ERS25269323	Metagenomic	Co-culture	Co-culture sample at day 3, pH 5, 8μg/ml (Figure 4)
DTU_2024_1036968_100_MG_4_5_8	ERS25269324	Metagenomic	Co-culture	Co-culture sample at day 4, pH 5, 8μg/ml (Figure 4)
DTU_2024_1036969_100_MG_5_5_8	ERS25269325	Metagenomic	Co-culture	Co-culture sample at day 5, pH 5, 8μg/ml (Figure 4)
DTU_2024_1036970_100_MG_2_5_16	ERS25269326	Metagenomic	Co-culture	Co-culture sample at day 2, pH 5, 16μg/ml (Figure 4)
DTU_2024_1036971_100_MG_3_5_16	ERS25269327	Metagenomic	Co-culture	Co-culture sample at day 3, pH 5, 16μg/ml (Figure 4)
DTU_2024_1036972_100_MG_4_5_16	ERS25269328	Metagenomic	Co-culture	Co-culture sample at day 4, pH 5, 16μg/ml (Figure 4)
DTU_2024_1036973_100_MG_5_5_16	ERS25269329	Metagenomic	Co-culture	Co-culture sample at day 5, pH 5, 16μg/ml (Figure 4)
DTU_2024_1036974_100_MG_2_8_4	ERS25269330	Metagenomic	Co-culture	Co-culture sample at day 2, pH 8, 4μg/ml (Figure 4)
DTU_2024_1036975_100_MG_3_8_4	ERS25269331	Metagenomic	Co-culture	Co-culture sample at day 3, pH 8, 4μg/ml (Figure 4)
DTU_2024_1036976_100_MG_4_8_4	ERS25269332	Metagenomic	Co-culture	Co-culture sample at day 4, pH 8, 4μg/ml (Figure 4)
DTU_2024_1036977_100_MG_5_8_4	ERS25269333	Metagenomic	Co-culture	Co-culture sample at day 5, pH 8, 4μg/ml (Figure 4)
DTU_2024_1036978_100_MG_2_8_8	ERS25269334	Metagenomic	Co-culture	Co-culture sample at day 2, pH 8, 8μg/ml (Figure 4)
DTU_2024_1036979_100_MG_3_8_8	ERS25269335	Metagenomic	Co-culture	Co-culture sample at day 3, pH 8, 8μg/ml (Figure 4)
DTU_2024_1036980_100_MG_4_8_8	ERS25269336	Metagenomic	Co-culture	Co-culture sample at day 4, pH 8, 8μg/ml (Figure 4)
DTU_2024_1036981_100_MG_5_8_8	ERS25269337	Metagenomic	Co-culture	Co-culture sample at day 5, pH 8, 8μg/ml (Figure 4)
DTU_2024_1036982_100_MG_2_8_16	ERS25269338	Metagenomic	Co-culture	Co-culture sample at day 2, pH 8, 16μg/ml (Figure 4)
DTU_2024_1036983_100_MG_3_8_16	ERS25269339	Metagenomic	Co-culture	Co-culture sample at day 3, pH 8, 16μg/ml (Figure 4)
DTU_2024_1036984_100_MG_4_8_16	ERS25269340	Metagenomic	Co-culture	Co-culture sample at day 4, pH 8, 16μg/ml (Figure 4)
DTU_2024_1036985_100_MG_5_8_16	ERS25269341	Metagenomic	Co-culture	Co-culture sample at day 5, pH 8, 16μg/ml (Figure 4)
DTU_2025_1037164_100_MG_2A1	ERS25269342	Metagenomic	Co-culture	Co-culture sample at 3h, constant pH 5, strains K12 CTX-M and K12 CMYCTX (Figure 5)
DTU_2025_1037165_100_MG_3A1	ERS25269343	Metagenomic	Co-culture	Co-culture sample at 6h, constant pH 5, strains K12 CTX-M and K12 CMYCTX (Figure 5)
DTU_2025_1037166_100_MG_4A1	ERS25269344	Metagenomic	Co-culture	Co-culture sample at 9h, constant pH 5, strains K12 CTX-M and K12 CMYCTX (Figure 5)
DTU_2025_1037167_100_MG_5A1	ERS25269345	Metagenomic	Co-culture	Co-culture sample at 12h, constant pH 5, strains K12 CTX-M and K12 CMYCTX (Figure 5)
DTU_2025_1037168_100_MG_6A1	ERS25269346	Metagenomic	Co-culture	Co-culture sample at 24h, constant pH 5, strains K12 CTX-M and K12 CMYCTX (Figure 5)
DTU_2025_1037169_100_MG_7A1	ERS25269347	Metagenomic	Co-culture	Co-culture sample at 36h, constant pH 5, strains K12 CTX-M and K12 CMYCTX (Figure 5)
DTU_2025_1037170_100_MG_8A1	ERS25269348	Metagenomic	Co-culture	Co-culture sample at 48h, constant pH 5, strains K12 CTX-M and K12 CMYCTX (Figure 5)
DTU_2025_1037172_100_MG_2A2	ERS25269349	Metagenomic	Co-culture	Co-culture sample at 3h, constant pH 5, strains K12 CMY and K12 CMYCTX (Figure 5)
DTU_2025_1037173_100_MG_3A2	ERS25269350	Metagenomic	Co-culture	Co-culture sample at 6h, constant pH 5, strains K12 CMY and K12 CMYCTX (Figure 5)
DTU_2025_1037174_100_MG_4A2	ERS25269351	Metagenomic	Co-culture	Co-culture sample at 9h, constant pH 5, strains K12 CMY and K12 CMYCTX (Figure 5)
DTU_2025_1037175_100_MG_5A2	ERS25269352	Metagenomic	Co-culture	Co-culture sample at 12h, constant pH 5, strains K12 CMY and K12 CMYCTX (Figure 5)
DTU_2025_1037176_100_MG_6A2	ERS25269353	Metagenomic	Co-culture	Co-culture sample at 24h, constant pH 5, strains K12 CMY and K12 CMYCTX (Figure 5)
DTU_2025_1037177_100_MG_7A2	ERS25269354	Metagenomic	Co-culture	Co-culture sample at 36h, constant pH 5, strains K12 CMY and K12 CMYCTX (Figure 5)
DTU_2025_1037178_100_MG_8A2	ERS25269355	Metagenomic	Co-culture	Co-culture sample at 48h, constant pH 5, strains K12 CMY and K12 CMYCTX (Figure 5)
DTU_2025_1037180_100_MG_2B1	ERS25269356	Metagenomic	Co-culture	Co-culture sample at 3h, constant pH 8, strains K12 CTX-M and K12 CMYCTX (Figure 5)
DTU_2025_1037181_100_MG_3B1	ERS25269357	Metagenomic	Co-culture	Co-culture sample at 6h, constant pH 8, strains K12 CTX-M and K12 CMYCTX (Figure 5)
DTU_2025_1037182_100_MG_4B1	ERS25269358	Metagenomic	Co-culture	Co-culture sample at 9h, constant pH 8, strains K12 CTX-M and K12 CMYCTX (Figure 5)
DTU_2025_1037183_100_MG_5B1	ERS25269359	Metagenomic	Co-culture	Co-culture sample at 12h, constant pH 8, strains K12 CTX-M and K12 CMYCTX (Figure 5)

DTU_2025_1037184_100_MG_6B1	ERS25269360	Metagenomic	Co-culture	Co-culture sample at 24h, constant pH 8, strains K12 CTX-M and K12 CMYCTX (Figure 5)
DTU_2025_1037185_100_MG_7B1	ERS25269361	Metagenomic	Co-culture	Co-culture sample at 36h, constant pH 8, strains K12 CTX-M and K12 CMYCTX (Figure 5)
DTU_2025_1037186_100_MG_8B1	ERS25269362	Metagenomic	Co-culture	Co-culture sample at 48h, constant pH 8, strains K12 CTX-M and K12 CMYCTX (Figure 5)
DTU_2025_1037188_100_MG_2B2	ERS25269363	Metagenomic	Co-culture	Co-culture sample at 3h, constant pH 8, strains K12 CMY and K12 CMYCTX (Figure 5)
DTU_2025_1037189_100_MG_3B2	ERS25269364	Metagenomic	Co-culture	Co-culture sample at 6h, constant pH 8, strains K12 CMY and K12 CMYCTX (Figure 5)
DTU_2025_1037190_100_MG_4B2	ERS25269365	Metagenomic	Co-culture	Co-culture sample at 9h, constant pH 8, strains K12 CMY and K12 CMYCTX (Figure 5)
DTU_2025_1037191_100_MG_5B2	ERS25269366	Metagenomic	Co-culture	Co-culture sample at 12h, constant pH 8, strains K12 CMY and K12 CMYCTX (Figure 5)
DTU_2025_1037192_100_MG_6B2	ERS25269367	Metagenomic	Co-culture	Co-culture sample at 24h, constant pH 8, strains K12 CMY and K12 CMYCTX (Figure 5)
DTU_2025_1037193_100_MG_7B2	ERS25269368	Metagenomic	Co-culture	Co-culture sample at 36h, constant pH 8, strains K12 CMY and K12 CMYCTX (Figure 5)
DTU_2025_1037194_100_MG_8B2	ERS25269369	Metagenomic	Co-culture	Co-culture sample at 48h, constant pH 8, strains K12 CMY and K12 CMYCTX (Figure 5)
DTU_2025_1037196_100_MG_2C1	ERS25269370	Metagenomic	Co-culture	Co-culture sample at 3h, RC start pH 5, strains K12 CTX-M and K12 CMYCTX (Figure 5)
DTU_2025_1037197_100_MG_3C1	ERS25269371	Metagenomic	Co-culture	Co-culture sample at 6h, RC start pH 5, strains K12 CTX-M and K12 CMYCTX (Figure 5)
DTU_2025_1037198_100_MG_4C1	ERS25269372	Metagenomic	Co-culture	Co-culture sample at 9h, RC start pH 5, strains K12 CTX-M and K12 CMYCTX (Figure 5)
DTU_2025_1037199_100_MG_5C1	ERS25269373	Metagenomic	Co-culture	Co-culture sample at 12h, RC start pH 5, strains K12 CTX-M and K12 CMYCTX (Figure 5)
DTU_2025_1037200_100_MG_6C1	ERS25269374	Metagenomic	Co-culture	Co-culture sample at 24h, RC start pH 5, strains K12 CTX-M and K12 CMYCTX (Figure 5)
DTU_2025_1037201_100_MG_7C1	ERS25269375	Metagenomic	Co-culture	Co-culture sample at 36h, RC start pH 5, strains K12 CTX-M and K12 CMYCTX (Figure 5)
DTU_2025_1037202_100_MG_8C1	ERS25269376	Metagenomic	Co-culture	Co-culture sample at 48h, RC start pH 5, strains K12 CTX-M and K12 CMYCTX (Figure 5)
DTU_2025_1037204_100_MG_2C2	ERS25269377	Metagenomic	Co-culture	Co-culture sample at 3h, RC start pH 5, strains K12 CMY and K12 CMYCTX (Figure 5)
DTU_2025_1037205_100_MG_3C2	ERS25269378	Metagenomic	Co-culture	Co-culture sample at 6h, RC start pH 5, strains K12 CMY and K12 CMYCTX (Figure 5)
DTU_2025_1037206_100_MG_4C2	ERS25269379	Metagenomic	Co-culture	Co-culture sample at 9h, RC start pH 5, strains K12 CMY and K12 CMYCTX (Figure 5)
DTU_2025_1037207_100_MG_5C2	ERS25269380	Metagenomic	Co-culture	Co-culture sample at 12h, RC start pH 5, strains K12 CMY and K12 CMYCTX (Figure 5)
DTU_2025_1037208_100_MG_6C2	ERS25269381	Metagenomic	Co-culture	Co-culture sample at 24h, RC start pH 5, strains K12 CMY and K12 CMYCTX (Figure 5)
DTU_2025_1037209_100_MG_7C2	ERS25269382	Metagenomic	Co-culture	Co-culture sample at 36h, RC start pH 5, strains K12 CMY and K12 CMYCTX (Figure 5)
DTU_2025_1037210_100_MG_8C2	ERS25269383	Metagenomic	Co-culture	Co-culture sample at 48h, RC start pH 5, strains K12 CMY and K12 CMYCTX (Figure 5)
DTU_2025_1037212_100_MG_2C3	ERS25269384	Metagenomic	Co-culture	Co-culture sample at 3h, RC start pH 5, strains K12 CTX-M and K12 CMY (Figure 5)
DTU_2025_1037213_100_MG_3C3	ERS25269385	Metagenomic	Co-culture	Co-culture sample at 6h, RC start pH 5, strains K12 CTX-M and K12 CMY (Figure 5)
DTU_2025_1037214_100_MG_4C3	ERS25269386	Metagenomic	Co-culture	Co-culture sample at 9h, RC start pH 5, strains K12 CTX-M and K12 CMY (Figure 5)
DTU_2025_1037215_100_MG_5C3	ERS25269387	Metagenomic	Co-culture	Co-culture sample at 12h, RC start pH 5, strains K12 CTX-M and K12 CMY (Figure 5)
DTU_2025_1037216_100_MG_6C3	ERS25269388	Metagenomic	Co-culture	Co-culture sample at 24h, RC start pH 5, strains K12 CTX-M and K12 CMY (Figure 5)
DTU_2025_1037217_100_MG_7C3	ERS25269389	Metagenomic	Co-culture	Co-culture sample at 36h, RC start pH 5, strains K12 CTX-M and K12 CMY (Figure 5)
DTU_2025_1037218_100_MG_8C3	ERS25269390	Metagenomic	Co-culture	Co-culture sample at 48h, RC start pH 5, strains K12 CTX-M and K12 CMY (Figure 5)
DTU_2025_1037220_100_MG_2D1	ERS25269391	Metagenomic	Co-culture	Co-culture sample at 3h, RC start pH 8, strains K12 CTX-M and K12 CMYCTX (Figure 5)
DTU_2025_1037221_100_MG_3D1	ERS25269392	Metagenomic	Co-culture	Co-culture sample at 6h, RC start pH 8, strains K12 CTX-M and K12 CMYCTX (Figure 5)
DTU_2025_1037222_100_MG_4D1	ERS25269393	Metagenomic	Co-culture	Co-culture sample at 9h, RC start pH 8, strains K12 CTX-M and K12 CMYCTX (Figure 5)
DTU_2025_1037223_100_MG_5D1	ERS25269394	Metagenomic	Co-culture	Co-culture sample at 12h, RC start pH 8, strains K12 CTX-M and K12 CMYCTX (Figure 5)
DTU_2025_1037224_100_MG_6D1	ERS25269395	Metagenomic	Co-culture	Co-culture sample at 24h, RC start pH 8, strains K12 CTX-M and K12 CMYCTX (Figure 5)
DTU_2025_1037225_100_MG_7D1	ERS25269396	Metagenomic	Co-culture	Co-culture sample at 36h, RC start pH 8, strains K12 CTX-M and K12 CMYCTX (Figure 5)
DTU_2025_1037226_100_MG_8D1	ERS25269397	Metagenomic	Co-culture	Co-culture sample at 48h, RC start pH 8, strains K12 CTX-M and K12 CMYCTX (Figure 5)
DTU_2025_1037228_100_MG_2D2	ERS25269398	Metagenomic	Co-culture	Co-culture sample at 3h, RC start pH 8, strains K12 CMY and K12 CMYCTX (Figure 5)
DTU_2025_1037229_100_MG_3D2	ERS25269399	Metagenomic	Co-culture	Co-culture sample at 6h, RC start pH 8, strains K12 CMY and K12 CMYCTX (Figure 5)
DTU_2025_1037230_100_MG_4D2	ERS25269400	Metagenomic	Co-culture	Co-culture sample at 9h, RC start pH 8, strains K12 CMY and K12 CMYCTX (Figure 5)
DTU_2025_1037231_100_MG_5D2	ERS25269401	Metagenomic	Co-culture	Co-culture sample at 12h, RC start pH 8, strains K12 CMY and K12 CMYCTX (Figure 5)
DTU_2025_1037232_100_MG_6D2	ERS25269402	Metagenomic	Co-culture	Co-culture sample at 24h, RC start pH 8, strains K12 CMY and K12 CMYCTX (Figure 5)
DTU_2025_1037233_100_MG_7D2	ERS25269403	Metagenomic	Co-culture	Co-culture sample at 36h, RC start pH 8, strains K12 CMY and K12 CMYCTX (Figure 5)
DTU_2025_1037234_100_MG_8D2	ERS25269404	Metagenomic	Co-culture	Co-culture sample at 48h, RC start pH 8, strains K12 CMY and K12 CMYCTX (Figure 5)
DTU_2025_1037236_100_MG_2D3	ERS25269405	Metagenomic	Co-culture	Co-culture sample at 3h, RC start pH 8, strains K12 CTX-M and K12 CMY (Figure 5)
DTU_2025_1037237_100_MG_3D3	ERS25269406	Metagenomic	Co-culture	Co-culture sample at 6h, RC start pH 8, strains K12 CTX-M and K12 CMY (Figure 5)

DTU_2025_1037238_100_MG_4D3	ERS25269407	Metagenomic	Co-culture	Co-culture sample at 9h, RC start pH 8, strains K12 CTX-M and K12 CMY (Figure 5)
DTU_2025_1037239_100_MG_5D3	ERS25269408	Metagenomic	Co-culture	Co-culture sample at 12h, RC start pH 8, strains K12 CTX-M and K12 CMY (Figure 5)
DTU_2025_1037240_100_MG_6D3	ERS25269409	Metagenomic	Co-culture	Co-culture sample at 24h, RC start pH 8, strains K12 CTX-M and K12 CMY (Figure 5)
DTU_2025_1037241_100_MG_7D3	ERS25269410	Metagenomic	Co-culture	Co-culture sample at 36h, RC start pH 8, strains K12 CTX-M and K12 CMY (Figure 5)
DTU_2025_1037242_100_MG_8D3	ERS25269411	Metagenomic	Co-culture	Co-culture sample at 48h, RC start pH 8, strains K12 CTX-M and K12 CMY (Figure 5)
DTU_2024_1036986_100_SI_K12_A	ERS25269412	Single isolate	Strain validation	K12 strain
DTU_2025_1037647_100_MG_1A1	ERS25269413	Metagenomic	Co-culture	Co-culture sample at 3h, Constant pH 5, strains K12 CTX-M and K12 CMYCTX (Figure 6)
DTU_2025_1037648_100_MG_2A1	ERS25269414	Metagenomic	Co-culture	Co-culture sample at 6h, Constant pH 5, strains K12 CTX-M and K12 CMYCTX (Figure 6)
DTU_2025_1037649_100_MG_3A1	ERS25269415	Metagenomic	Co-culture	Co-culture sample at 9h, Constant pH 5, strains K12 CTX-M and K12 CMYCTX (Figure 6)
DTU_2025_1037650_100_MG_4A1	ERS25269416	Metagenomic	Co-culture	Co-culture sample at 12h, Constant pH 5, strains K12 CTX-M and K12 CMYCTX (Figure 6)
DTU_2025_1037651_100_MG_1A2	ERS25269417	Metagenomic	Co-culture	Co-culture sample at 3h, Constant pH 5, strains K12 CMY and K12 CMYCTX (Figure 6)
DTU_2025_1037652_100_MG_2A2	ERS25269418	Metagenomic	Co-culture	Co-culture sample at 6h, Constant pH 5, strains K12 CMY and K12 CMYCTX (Figure 6)
DTU_2025_1037653_100_MG_3A2	ERS25269419	Metagenomic	Co-culture	Co-culture sample at 9h, Constant pH 5, strains K12 CMY and K12 CMYCTX (Figure 6)
DTU_2025_1037654_100_MG_4A2	ERS25269420	Metagenomic	Co-culture	Co-culture sample at 12h, Constant pH 5, strains K12 CMY and K12 CMYCTX (Figure 6)
DTU_2025_1037655_100_MG_1B1	ERS25269421	Metagenomic	Co-culture	Co-culture sample at 3h, Constant pH 8, strains K12 CTX-M and K12 CMYCTX (Figure 6)
DTU_2025_1037656_100_MG_2B1	ERS25269422	Metagenomic	Co-culture	Co-culture sample at 6h, Constant pH 8, strains K12 CTX-M and K12 CMYCTX (Figure 6)
DTU_2025_1037657_100_MG_3B1	ERS25269423	Metagenomic	Co-culture	Co-culture sample at 9h, Constant pH 8, strains K12 CTX-M and K12 CMYCTX (Figure 6)
DTU_2025_1037658_100_MG_4B1	ERS25269424	Metagenomic	Co-culture	Co-culture sample at 12h, Constant pH 8, strains K12 CTX-M and K12 CMYCTX (Figure 6)
DTU_2025_1037659_100_MG_1B2	ERS25269425	Metagenomic	Co-culture	Co-culture sample at 3h, Constant pH 8, strains K12 CMY and K12 CMYCTX (Figure 6)
DTU_2025_1037660_100_MG_2B2	ERS25269426	Metagenomic	Co-culture	Co-culture sample at 6h, Constant pH 8, strains K12 CMY and K12 CMYCTX (Figure 6)
DTU_2025_1037661_100_MG_3B2	ERS25269427	Metagenomic	Co-culture	Co-culture sample at 9h, Constant pH 8, strains K12 CMY and K12 CMYCTX (Figure 6)
DTU_2025_1037662_100_MG_4B2	ERS25269428	Metagenomic	Co-culture	Co-culture sample at 12h, Constant pH 8, strains K12 CMY and K12 CMYCTX (Figure 6)
DTU_2025_1037663_100_MG_1C1	ERS25269429	Metagenomic	Co-culture	Co-culture sample at 3h, RC start pH 5, strains K12 CTX-M and K12 CMYCTX (Figure 6)
DTU_2025_1037664_100_MG_2C1	ERS25269430	Metagenomic	Co-culture	Co-culture sample at 6h, RC start pH 5, strains K12 CTX-M and K12 CMYCTX (Figure 6)
DTU_2025_1037665_100_MG_3C1	ERS25269431	Metagenomic	Co-culture	Co-culture sample at 9h, RC start pH 5, strains K12 CTX-M and K12 CMYCTX (Figure 6)
DTU_2025_1037666_100_MG_4C1	ERS25269432	Metagenomic	Co-culture	Co-culture sample at 12h, RC start pH 5, strains K12 CTX-M and K12 CMYCTX (Figure 6)
DTU_2025_1037667_100_MG_1C2	ERS25269433	Metagenomic	Co-culture	Co-culture sample at 3h, RC start pH 5, strains K12 CMY and K12 CMYCTX (Figure 6)
DTU_2025_1037668_100_MG_2C2	ERS25269434	Metagenomic	Co-culture	Co-culture sample at 6h, RC start pH 5, strains K12 CMY and K12 CMYCTX (Figure 6)
DTU_2025_1037669_100_MG_3C2	ERS25269435	Metagenomic	Co-culture	Co-culture sample at 9h, RC start pH 5, strains K12 CMY and K12 CMYCTX (Figure 6)
DTU_2025_1037670_100_MG_4C2	ERS25269436	Metagenomic	Co-culture	Co-culture sample at 12h, RC start pH 5, strains K12 CMY and K12 CMYCTX (Figure 6)
DTU_2025_1037671_100_MG_1C3	ERS25269437	Metagenomic	Co-culture	Co-culture sample at 3h, RC start pH 5, strains K12 CTX-M and K12 CMY (Figure 6)
DTU_2025_1037672_100_MG_2C3	ERS25269438	Metagenomic	Co-culture	Co-culture sample at 6h, RC start pH 5, strains K12 CTX-M and K12 CMY (Figure 6)
DTU_2025_1037673_100_MG_3C3	ERS25269439	Metagenomic	Co-culture	Co-culture sample at 9h, RC start pH 5, strains K12 CTX-M and K12 CMY (Figure 6)
DTU_2025_1037674_100_MG_1D1	ERS25269440	Metagenomic	Co-culture	Co-culture sample at 3h, RC start pH 8, strains K12 CTX-M and K12 CMYCTX (Figure 6)
DTU_2025_1037675_100_MG_2D1	ERS25269441	Metagenomic	Co-culture	Co-culture sample at 6h, RC start pH 8, strains K12 CTX-M and K12 CMYCTX (Figure 6)
DTU_2025_1037676_100_MG_3D1	ERS25269442	Metagenomic	Co-culture	Co-culture sample at 9h, RC start pH 8, strains K12 CTX-M and K12 CMYCTX (Figure 6)
DTU_2025_1037677_100_MG_4D1	ERS25269443	Metagenomic	Co-culture	Co-culture sample at 12h, RC start pH 8, strains K12 CTX-M and K12 CMYCTX (Figure 6)
DTU_2025_1037678_100_MG_1D2	ERS25269444	Metagenomic	Co-culture	Co-culture sample at 3h, RC start pH 8, strains K12 CMY and K12 CMYCTX (Figure 6)
DTU_2025_1037679_100_MG_2D2	ERS25269445	Metagenomic	Co-culture	Co-culture sample at 6h, RC start pH 8, strains K12 CMY and K12 CMYCTX (Figure 6)
DTU_2025_1037680_100_MG_3D2	ERS25269446	Metagenomic	Co-culture	Co-culture sample at 9h, RC start pH 8, strains K12 CMY and K12 CMYCTX (Figure 6)
DTU_2025_1037681_100_MG_4D2	ERS25269447	Metagenomic	Co-culture	Co-culture sample at 12h, RC start pH 8, strains K12 CMY and K12 CMYCTX (Figure 6)
DTU_2025_1037682_100_MG_1D3	ERS25269448	Metagenomic	Co-culture	Co-culture sample at 3h, RC start pH 8, strains K12 CTX-M and K12 CMY (Figure 6)
DTU_2025_1037683_100_MG_2D3	ERS25269449	Metagenomic	Co-culture	Co-culture sample at 6h, RC start pH 8, strains K12 CTX-M and K12 CMY (Figure 6)
DTU_2025_1037684_100_MG_3D3	ERS25269450	Metagenomic	Co-culture	Co-culture sample at 9h, RC start pH 8, strains K12 CTX-M and K12 CMY (Figure 6)
DTU_2025_1037685_100_MG_4D3	ERS25269451	Metagenomic	Co-culture	Co-culture sample at 12h, RC start pH 8, strains K12 CTX-M and K12 CMY (Figure 6)
DTU_2025_1037688_100_SI_MA146	ERS25269452	Single isolate	Strain validation	K12 CMYCTX strain
DTU_2025_1038607_1_MG_1A1_notaz_3h_SE	ERS27209483	Metagenomic	Co-culture	Co-culture sample at 3h, Constant pH 5, strains K12 CTX-M and K12 CMYCTX No ceftazidime, (Figure S 9)

DTU_2025_1038608_1_MG_2A1_notaz_6h_SE	ERS27209484	Metagenomic	Co-culture	Co-culture sample at 6h, Constant pH 5, strains K12 CTX-M and K12 CMYCTX No ceftazidime, (Figure S 9)
DTU_2025_1038609_1_MG_3A1_notaz_9h_SE	ERS27209485	Metagenomic	Co-culture	Co-culture sample at 9h, Constant pH 5, strains K12 CTX-M and K12 CMYCTX No ceftazidime, (Figure S 9)
DTU_2025_1038610_1_MG_4A1_notaz_12h_SE	ERS27209486	Metagenomic	Co-culture	Co-culture sample at 12h, Constant pH 5, strains K12 CTX-M and K12 CMYCTX No ceftazidime, (Figure S 9)
DTU_2025_1038611_1_MG_1A2_notaz_3h_SE	ERS27209487	Metagenomic	Co-culture	Co-culture sample at 3h, Constant pH 5, strains K12 CMY and K12 CMYCTX No ceftazidime, (Figure S 9)
DTU_2025_1038612_1_MG_2A2_notaz_6h_SE	ERS27209488	Metagenomic	Co-culture	Co-culture sample at 6h, Constant pH 5, strains K12 CMY and K12 CMYCTX No ceftazidime, (Figure S 9)
DTU_2025_1038613_1_MG_3A2_notaz_9h_SE	ERS27209489	Metagenomic	Co-culture	Co-culture sample at 9h, Constant pH 5, strains K12 CMY and K12 CMYCTX No ceftazidime, (Figure S 9)
DTU_2025_1038614_1_MG_4A2_notaz_12h_SE	ERS27209490	Metagenomic	Co-culture	Co-culture sample at 12h, Constant pH 5, strains K12 CMY and K12 CMYCTX No ceftazidime, (Figure S 9)
DTU_2025_1038615_1_MG_1B1_notaz_3h_SE	ERS27209491	Metagenomic	Co-culture	Co-culture sample at 3h, Constant pH 8, strains K12 CTX-M and K12 CMYCTX No ceftazidime, (Figure S 9)
DTU_2025_1038616_1_MG_2B1_notaz_6h_SE	ERS27209492	Metagenomic	Co-culture	Co-culture sample at 6h, Constant pH 8, strains K12 CTX-M and K12 CMYCTX No ceftazidime, (Figure S 9)
DTU_2025_1038617_1_MG_3B1_notaz_9h_SE	ERS27209493	Metagenomic	Co-culture	Co-culture sample at 9h, Constant pH 8, strains K12 CTX-M and K12 CMYCTX No ceftazidime, (Figure S 9)
DTU_2025_1038618_1_MG_4B1_notaz_12h_SE	ERS27209494	Metagenomic	Co-culture	Co-culture sample at 12h, Constant pH 8, strains K12 CTX-M and K12 CMYCTX No ceftazidime, (Figure S 9)
DTU_2025_1038619_1_MG_1B2_notaz_3h_SE	ERS27209495	Metagenomic	Co-culture	Co-culture sample at 3h, Constant pH 8, strains K12 CMY and K12 CMYCTX No ceftazidime, (Figure S 9)
DTU_2025_1038620_1_MG_2B2_notaz_6h_SE	ERS27209496	Metagenomic	Co-culture	Co-culture sample at 6h, Constant pH 8, strains K12 CMY and K12 CMYCTX No ceftazidime, (Figure S 9)
DTU_2025_1038621_1_MG_3B2_notaz_9h_SE	ERS27209497	Metagenomic	Co-culture	Co-culture sample at 9h, Constant pH 8, strains K12 CMY and K12 CMYCTX No ceftazidime, (Figure S 9)
DTU_2025_1038622_1_MG_4B2_notaz_12h_SE	ERS27209498	Metagenomic	Co-culture	Co-culture sample at 12h, Constant pH 8, strains K12 CMY and K12 CMYCTX No ceftazidime, (Figure S 9)
DTU_2025_1038623_1_MG_1C1_notaz_3h_SE	ERS27209499	Metagenomic	Co-culture	Co-culture sample at 3h, RC start pH 5, strains K12 CTX-M and K12 CMYCTX No ceftazidime, (Figure S 9)
DTU_2025_1038624_1_MG_2C1_notaz_6h_SE	ERS27209500	Metagenomic	Co-culture	Co-culture sample at 6h, RC start pH 5, strains K12 CTX-M and K12 CMYCTX No ceftazidime, (Figure S 9)
DTU_2025_1038625_1_MG_3C1_notaz_9h_SE	ERS27209501	Metagenomic	Co-culture	Co-culture sample at 9h, RC start pH 5, strains K12 CTX-M and K12 CMYCTX No ceftazidime, (Figure S 9)
DTU_2025_1038626_1_MG_4C1_notaz_12h_SE	ERS27209502	Metagenomic	Co-culture	Co-culture sample at 12h, RC start pH 5, strains K12 CTX-M and K12 CMYCTX No ceftazidime, (Figure S 9)
DTU_2025_1038627_1_MG_1C2_notaz_3h_SE	ERS27209503	Metagenomic	Co-culture	Co-culture sample at 3h, RC start pH 5, strains K12 CMY and K12 CMYCTX No ceftazidime, (Figure S 9)
DTU_2025_1038628_1_MG_2C2_notaz_6h_SE	ERS27209504	Metagenomic	Co-culture	Co-culture sample at 6h, RC start pH 5, strains K12 CMY and K12 CMYCTX No ceftazidime, (Figure S 9)
DTU_2025_1038629_1_MG_3C2_notaz_9h_SE	ERS27209505	Metagenomic	Co-culture	Co-culture sample at 9h, RC start pH 5, strains K12 CMY and K12 CMYCTX No ceftazidime, (Figure S 9)
DTU_2025_1038630_1_MG_4C2_notaz_12h_SE	ERS27209506	Metagenomic	Co-culture	Co-culture sample at 12h, RC start pH 5, strains K12 CMY and K12 CMYCTX No ceftazidime, (Figure S 9)
DTU_2025_1038631_1_MG_1C3_notaz_3h_SE	ERS27209507	Metagenomic	Co-culture	Co-culture sample at 3h, RC start pH 5, strains K12 CTX-M and K12 CMY No ceftazidime, (Figure S 9)
DTU_2025_1038632_1_MG_2C3_notaz_6h_SE	ERS27209508	Metagenomic	Co-culture	Co-culture sample at 6h, RC start pH 5, strains K12 CTX-M and K12 CMY No ceftazidime, (Figure S 9)
DTU_2025_1038633_1_MG_3C3_notaz_9h_SE	ERS27209509	Metagenomic	Co-culture	Co-culture sample at 9h, RC start pH 5, strains K12 CTX-M and K12 CMY No ceftazidime, (Figure S 9)
DTU_2025_1038634_1_MG_4C3_notaz_12h_SE	ERS27209510	Metagenomic	Co-culture	Co-culture sample at 12h, RC start pH 5, strains K12 CTX-M and K12 CMY No ceftazidime, (Figure S 9)
DTU_2025_1038635_1_MG_1D1_notaz_3h_SE	ERS27209511	Metagenomic	Co-culture	Co-culture sample at 3h, RC start pH 8, strains K12 CTX-M and K12 CMYCTX No ceftazidime, (Figure S 9)
DTU_2025_1038636_1_MG_2D1_notaz_6h_SE	ERS27209512	Metagenomic	Co-culture	Co-culture sample at 6h, RC start pH 8, strains K12 CTX-M and K12 CMYCTX No ceftazidime, (Figure S 9)
DTU_2025_1038637_1_MG_3D1_notaz_9h_SE	ERS27209513	Metagenomic	Co-culture	Co-culture sample at 9h, RC start pH 8, strains K12 CTX-M and K12 CMYCTX No ceftazidime, (Figure S 9)
DTU_2025_1038638_1_MG_4D1_notaz_12h_SE	ERS27209514	Metagenomic	Co-culture	Co-culture sample at 12h, RC start pH 8, strains K12 CTX-M and K12 CMYCTX No ceftazidime, (Figure S 9)
DTU_2025_1038639_1_MG_1D2_notaz_3h_SE	ERS27209515	Metagenomic	Co-culture	Co-culture sample at 3h, RC start pH 8, strains K12 CMY and K12 CMYCTX No ceftazidime, (Figure S 9)
DTU_2025_1038640_1_MG_2D2_notaz_6h_SE	ERS27209516	Metagenomic	Co-culture	Co-culture sample at 6h, RC start pH 8, strains K12 CMY and K12 CMYCTX No ceftazidime, (Figure S 9)
DTU_2025_1038641_1_MG_3D2_notaz_9h_SE	ERS27209517	Metagenomic	Co-culture	Co-culture sample at 9h, RC start pH 8, strains K12 CMY and K12 CMYCTX No ceftazidime, (Figure S 9)
DTU_2025_1038642_1_MG_4D2_notaz_12h_SE	ERS27209518	Metagenomic	Co-culture	Co-culture sample at 12h, RC start pH 8, strains K12 CMY and K12 CMYCTX No ceftazidime, (Figure S 9)
DTU_2025_1038643_1_MG_1D3_notaz_3h_SE	ERS27209519	Metagenomic	Co-culture	Co-culture sample at 3h, RC start pH 8, strains K12 CTX-M and K12 CMY No ceftazidime, (Figure S 9)
DTU_2025_1038644_1_MG_2D3_notaz_6h_SE	ERS27209520	Metagenomic	Co-culture	Co-culture sample at 6h, RC start pH 8, strains K12 CTX-M and K12 CMY No ceftazidime, (Figure S 9)
DTU_2025_1038645_1_MG_3D3_notaz_9h_SE	ERS27209521	Metagenomic	Co-culture	Co-culture sample at 9h, RC start pH 8, strains K12 CTX-M and K12 CMY No ceftazidime, (Figure S 9)
DTU_2025_1038646_1_MG_4D3_notaz_12h_SE	ERS27209522	Metagenomic	Co-culture	Co-culture sample at 12h, RC start pH 8, strains K12 CTX-M and K12 CMY No ceftazidime, (Figure S 9)
DTU_2025_1038727_1_MG_2_5_0_notaz_24h	ERS27954614	Metagenomic	Co-culture	Co-culture sample at day 2, pH 5, no ceftazidime (Figure S 7)
DTU_2025_1038728_1_MG_3_5_0_notaz_48h	ERS27954615	Metagenomic	Co-culture	Co-culture sample at day 3, pH 5, no ceftazidime (Figure S 7)
DTU_2025_1038729_1_MG_4_5_0_notaz_72h	ERS27954616	Metagenomic	Co-culture	Co-culture sample at day 4, pH 5, no ceftazidime (Figure S 7)
DTU_2025_1038730_1_MG_5_5_0_notaz_96h	ERS27954617	Metagenomic	Co-culture	Co-culture sample at day 5, pH 5, no ceftazidime (Figure S 7)
DTU_2025_1038731_1_MG_2_8_0_notaz_24h	ERS27954618	Metagenomic	Co-culture	Co-culture sample at day 2, pH 8, no ceftazidime (Figure S 7)
DTU_2025_1038732_1_MG_3_8_0_notaz_48h	ERS27954619	Metagenomic	Co-culture	Co-culture sample at day 3, pH 8, no ceftazidime (Figure S 7)
DTU_2025_1038733_1_MG_4_8_0_notaz_72h	ERS27954620	Metagenomic	Co-culture	Co-culture sample at day 4, pH 8, no ceftazidime (Figure S 7)
DTU_2025_1038734_1_MG_5_8_0_notaz_96h	ERS27954621	Metagenomic	Co-culture	Co-culture sample at day 5, pH 8, no ceftazidime (Figure S 7)

DTU_2025_1038647_1_MG_1A1_notaz_0h	ERS27954622	Metagenomic	Co-culture	Co-culture sample at 0h, constant pH 5, strains K12 CTX-M and K12 CMYCTX No ceftazidime, (Figure S 8)
DTU_2025_1038648_1_MG_2A1_notaz_3h	ERS27954623	Metagenomic	Co-culture	Co-culture sample at 3h, constant pH 5, strains K12 CTX-M and K12 CMYCTX No ceftazidime, (Figure S 8)
DTU_2025_1038649_1_MG_3A1_notaz_6h	ERS27954624	Metagenomic	Co-culture	Co-culture sample at 6h, constant pH 5, strains K12 CTX-M and K12 CMYCTX No ceftazidime, (Figure S 8)
DTU_2025_1038650_1_MG_4A1_notaz_9h	ERS27954625	Metagenomic	Co-culture	Co-culture sample at 9h, constant pH 5, strains K12 CTX-M and K12 CMYCTX No ceftazidime, (Figure S 8)
DTU_2025_1038651_1_MG_5A1_notaz_12h	ERS27954626	Metagenomic	Co-culture	Co-culture sample at 12h, constant pH 5, strains K12 CTX-M and K12 CMYCTX No ceftazidime, (Figure S 8)
DTU_2025_1038652_1_MG_6A1_notaz_24h	ERS27954627	Metagenomic	Co-culture	Co-culture sample at 24h, constant pH 5, strains K12 CTX-M and K12 CMYCTX No ceftazidime, (Figure S 8)
DTU_2025_1038653_1_MG_7A1_notaz_36h	ERS27954628	Metagenomic	Co-culture	Co-culture sample at 36h, constant pH 5, strains K12 CTX-M and K12 CMYCTX No ceftazidime, (Figure S 8)
DTU_2025_1038654_1_MG_8A1_notaz_48h	ERS27954629	Metagenomic	Co-culture	Co-culture sample at 48h, constant pH 5, strains K12 CTX-M and K12 CMYCTX No ceftazidime, (Figure S 8)
DTU_2025_1038655_1_MG_1B1_notaz_0h	ERS27954630	Metagenomic	Co-culture	Co-culture sample at 0h, constant pH 8, strains K12 CTX-M and K12 CMYCTX No ceftazidime, (Figure S 8)
DTU_2025_1038656_1_MG_2B1_notaz_3h	ERS27954631	Metagenomic	Co-culture	Co-culture sample at 3h, constant pH 8, strains K12 CTX-M and K12 CMYCTX No ceftazidime, (Figure S 8)
DTU_2025_1038657_1_MG_3B1_notaz_6h	ERS27954632	Metagenomic	Co-culture	Co-culture sample at 6h, constant pH 8, strains K12 CTX-M and K12 CMYCTX No ceftazidime, (Figure S 8)
DTU_2025_1038658_1_MG_4B1_notaz_9h	ERS27954633	Metagenomic	Co-culture	Co-culture sample at 9h, constant pH 8, strains K12 CTX-M and K12 CMYCTX No ceftazidime, (Figure S 8)
DTU_2025_1038659_1_MG_5B1_notaz_12h	ERS27954634	Metagenomic	Co-culture	Co-culture sample at 12h, constant pH 8, strains K12 CTX-M and K12 CMYCTX No ceftazidime, (Figure S 8)
DTU_2025_1038660_1_MG_6B1_notaz_24h	ERS27954635	Metagenomic	Co-culture	Co-culture sample at 24h, constant pH 8, strains K12 CTX-M and K12 CMYCTX No ceftazidime, (Figure S 8)
DTU_2025_1038661_1_MG_7B1_notaz_36h	ERS27954636	Metagenomic	Co-culture	Co-culture sample at 36h, constant pH 8, strains K12 CTX-M and K12 CMYCTX No ceftazidime, (Figure S 8)
DTU_2025_1038662_1_MG_8B1_notaz_48h	ERS27954637	Metagenomic	Co-culture	Co-culture sample at 48h, constant pH 8, strains K12 CTX-M and K12 CMYCTX No ceftazidime, (Figure S 8)
DTU_2025_1038663_1_MG_1C1_notaz_0h	ERS27954638	Metagenomic	Co-culture	Co-culture sample at 0h, RC start pH 5, strains K12 CTX-M and K12 CMYCTX No ceftazidime, (Figure S 8)
DTU_2025_1038664_1_MG_2C1_notaz_3h	ERS27954639	Metagenomic	Co-culture	Co-culture sample at 3h, RC start pH 5, strains K12 CTX-M and K12 CMYCTX No ceftazidime, (Figure S 8)
DTU_2025_1038665_1_MG_3C1_notaz_6h	ERS27954640	Metagenomic	Co-culture	Co-culture sample at 6h, RC start pH 5, strains K12 CTX-M and K12 CMYCTX No ceftazidime, (Figure S 8)
DTU_2025_1038666_1_MG_4C1_notaz_9h	ERS27954641	Metagenomic	Co-culture	Co-culture sample at 9h, RC start pH 5, strains K12 CTX-M and K12 CMYCTX No ceftazidime, (Figure S 8)
DTU_2025_1038667_1_MG_5C1_notaz_12h	ERS27954642	Metagenomic	Co-culture	Co-culture sample at 12h, RC start pH 5, strains K12 CTX-M and K12 CMYCTX No ceftazidime, (Figure S 8)
DTU_2025_1038668_1_MG_6C1_notaz_24h	ERS27954643	Metagenomic	Co-culture	Co-culture sample at 24h, RC start pH 5, strains K12 CTX-M and K12 CMYCTX No ceftazidime, (Figure S 8)
DTU_2025_1038669_1_MG_7C1_notaz_36h	ERS27954644	Metagenomic	Co-culture	Co-culture sample at 36h, RC start pH 5, strains K12 CTX-M and K12 CMYCTX No ceftazidime, (Figure S 8)
DTU_2025_1038670_1_MG_8C1_notaz_48h	ERS27954645	Metagenomic	Co-culture	Co-culture sample at 48h, RC start pH 5, strains K12 CTX-M and K12 CMYCTX No ceftazidime, (Figure S 8)
DTU_2025_1038671_1_MG_1D1_notaz_0h	ERS27954647	Metagenomic	Co-culture	Co-culture sample at 0h, RC start pH 8, strains K12 CTX-M and K12 CMYCTX No ceftazidime, (Figure S 8)
DTU_2025_1038672_1_MG_2D1_notaz_3h	ERS27954648	Metagenomic	Co-culture	Co-culture sample at 3h, RC start pH 8, strains K12 CTX-M and K12 CMYCTX No ceftazidime, (Figure S 8)
DTU_2025_1038673_1_MG_3D1_notaz_6h	ERS27954649	Metagenomic	Co-culture	Co-culture sample at 6h, RC start pH 8, strains K12 CTX-M and K12 CMYCTX No ceftazidime, (Figure S 8)
DTU_2025_1038674_1_MG_4D1_notaz_9h	ERS27954650	Metagenomic	Co-culture	Co-culture sample at 9h, RC start pH 8, strains K12 CTX-M and K12 CMYCTX No ceftazidime, (Figure S 8)
DTU_2025_1038675_1_MG_5D1_notaz_12h	ERS27954651	Metagenomic	Co-culture	Co-culture sample at 12h, RC start pH 8, strains K12 CTX-M and K12 CMYCTX No ceftazidime, (Figure S 8)
DTU_2025_1038676_1_MG_6D1_notaz_24h	ERS27954652	Metagenomic	Co-culture	Co-culture sample at 24h, RC start pH 8, strains K12 CTX-M and K12 CMYCTX No ceftazidime, (Figure S 8)
DTU_2025_1038677_1_MG_7D1_notaz_36h	ERS27954653	Metagenomic	Co-culture	Co-culture sample at 36h, RC start pH 8, strains K12 CTX-M and K12 CMYCTX No ceftazidime, (Figure S 8)
DTU_2025_1038678_1_MG_8D1_notaz_48h	ERS27954654	Metagenomic	Co-culture	Co-culture sample at 48h, RC start pH 8, strains K12 CTX-M and K12 CMYCTX No ceftazidime, (Figure S 8)
DTU_2025_1038679_1_MG_1A2_notaz_0h	ERS27954655	Metagenomic	Co-culture	Co-culture sample at 0h, constant pH 5, strains K12 CMY and K12 CMYCTX No ceftazidime, (Figure S 8)
DTU_2025_1038680_1_MG_2A2_notaz_3h	ERS27954656	Metagenomic	Co-culture	Co-culture sample at 3h, constant pH 5, strains K12 CMY and K12 CMYCTX No ceftazidime, (Figure S 8)
DTU_2025_1038681_1_MG_3A2_notaz_6h	ERS27954657	Metagenomic	Co-culture	Co-culture sample at 6h, constant pH 5, strains K12 CMY and K12 CMYCTX No ceftazidime, (Figure S 8)
DTU_2025_1038682_1_MG_4A2_notaz_9h	ERS27954658	Metagenomic	Co-culture	Co-culture sample at 9h, constant pH 5, strains K12 CMY and K12 CMYCTX No ceftazidime, (Figure S 8)
DTU_2025_1038683_1_MG_5A2_notaz_12h	ERS27954659	Metagenomic	Co-culture	Co-culture sample at 12h, constant pH 5, strains K12 CMY and K12 CMYCTX No ceftazidime, (Figure S 8)
DTU_2025_1038684_1_MG_6A2_notaz_24h	ERS27954660	Metagenomic	Co-culture	Co-culture sample at 24h, constant pH 5, strains K12 CMY and K12 CMYCTX No ceftazidime, (Figure S 8)
DTU_2025_1038685_1_MG_7A2_notaz_36h	ERS27954661	Metagenomic	Co-culture	Co-culture sample at 36h, constant pH 5, strains K12 CMY and K12 CMYCTX No ceftazidime, (Figure S 8)
DTU_2025_1038686_1_MG_8A2_notaz_48h	ERS27954662	Metagenomic	Co-culture	Co-culture sample at 48h, constant pH 5, strains K12 CMY and K12 CMYCTX No ceftazidime, (Figure S 8)
DTU_2025_1038687_1_MG_1B2_notaz_0h	ERS27954663	Metagenomic	Co-culture	Co-culture sample at 0h, constant pH 8, strains K12 CMY and K12 CMYCTX No ceftazidime, (Figure S 8)
DTU_2025_1038688_1_MG_2B2_notaz_3h	ERS27954664	Metagenomic	Co-culture	Co-culture sample at 3h, constant pH 8, strains K12 CMY and K12 CMYCTX No ceftazidime, (Figure S 8)
DTU_2025_1038689_1_MG_3B2_notaz_6h	ERS27954665	Metagenomic	Co-culture	Co-culture sample at 6h, constant pH 8, strains K12 CMY and K12 CMYCTX No ceftazidime, (Figure S 8)
DTU_2025_1038690_1_MG_4B2_notaz_9h	ERS27954666	Metagenomic	Co-culture	Co-culture sample at 9h, constant pH 8, strains K12 CMY and K12 CMYCTX No ceftazidime, (Figure S 8)
DTU_2025_1038691_1_MG_5B2_notaz_12h	ERS27954667	Metagenomic	Co-culture	Co-culture sample at 12h, constant pH 8, strains K12 CMY and K12 CMYCTX No ceftazidime, (Figure S 8)
DTU_2025_1038692_1_MG_6B2_notaz_24h	ERS27954668	Metagenomic	Co-culture	Co-culture sample at 24h, constant pH 8, strains K12 CMY and K12 CMYCTX No ceftazidime, (Figure S 8)
DTU_2025_1038693_1_MG_7B2_notaz_36h	ERS27954669	Metagenomic	Co-culture	Co-culture sample at 36h, constant pH 8, strains K12 CMY and K12 CMYCTX No ceftazidime, (Figure S 8)

DTU_2025_1038694_1_MG_8B2_notaz_48h	ERS27954670	Metagenomic	Co-culture	Co-culture sample at 48h, constant pH 8, strains K12 CMY and K12 CMYCTX No ceftazidime, (Figure S 8)
DTU_2025_1038695_1_MG_1C2_notaz_0h	ERS27954671	Metagenomic	Co-culture	Co-culture sample at 0h, RC start pH 5, strains K12 CMY and K12 CMYCTX No ceftazidime, (Figure S 8)
DTU_2025_1038696_1_MG_2C2_notaz_3h	ERS27954672	Metagenomic	Co-culture	Co-culture sample at 3h, RC start pH 5, strains K12 CMY and K12 CMYCTX No ceftazidime, (Figure S 8)
DTU_2025_1038697_1_MG_3C2_notaz_6h	ERS27954673	Metagenomic	Co-culture	Co-culture sample at 6h, RC start pH 5, strains K12 CMY and K12 CMYCTX No ceftazidime, (Figure S 8)
DTU_2025_1038698_1_MG_4C2_notaz_9h	ERS27954674	Metagenomic	Co-culture	Co-culture sample at 9h, RC start pH 5, strains K12 CMY and K12 CMYCTX No ceftazidime, (Figure S 8)
DTU_2025_1038699_1_MG_5C2_notaz_12h	ERS27954675	Metagenomic	Co-culture	Co-culture sample at 12h, RC start pH 5, strains K12 CMY and K12 CMYCTX No ceftazidime, (Figure S 8)
DTU_2025_1038700_1_MG_6C2_notaz_24h	ERS27954676	Metagenomic	Co-culture	Co-culture sample at 24h, RC start pH 5, strains K12 CMY and K12 CMYCTX No ceftazidime, (Figure S 8)
DTU_2025_1038701_1_MG_7C2_notaz_36h	ERS27954677	Metagenomic	Co-culture	Co-culture sample at 36h, RC start pH 5, strains K12 CMY and K12 CMYCTX No ceftazidime, (Figure S 8)
DTU_2025_1038702_1_MG_8C2_notaz_48h	ERS27954678	Metagenomic	Co-culture	Co-culture sample at 48h, RC start pH 5, strains K12 CMY and K12 CMYCTX No ceftazidime, (Figure S 8)
DTU_2025_1038703_1_MG_1D2_notaz_0h	ERS27954679	Metagenomic	Co-culture	Co-culture sample at 0h, RC start pH 8, strains K12 CMY and K12 CMYCTX No ceftazidime, (Figure S 8)
DTU_2025_1038704_1_MG_2D2_notaz_3h	ERS27954680	Metagenomic	Co-culture	Co-culture sample at 3h, RC start pH 8, strains K12 CMY and K12 CMYCTX No ceftazidime, (Figure S 8)
DTU_2025_1038705_1_MG_3D2_notaz_6h	ERS27954681	Metagenomic	Co-culture	Co-culture sample at 6h, RC start pH 8, strains K12 CMY and K12 CMYCTX No ceftazidime, (Figure S 8)
DTU_2025_1038706_1_MG_4D2_notaz_9h	ERS27954682	Metagenomic	Co-culture	Co-culture sample at 9h, RC start pH 8, strains K12 CMY and K12 CMYCTX No ceftazidime, (Figure S 8)
DTU_2025_1038707_1_MG_5D2_notaz_12h	ERS27954683	Metagenomic	Co-culture	Co-culture sample at 12h, RC start pH 8, strains K12 CMY and K12 CMYCTX No ceftazidime, (Figure S 8)
DTU_2025_1038708_1_MG_6D2_notaz_24h	ERS27954684	Metagenomic	Co-culture	Co-culture sample at 24h, RC start pH 8, strains K12 CMY and K12 CMYCTX No ceftazidime, (Figure S 8)
DTU_2025_1038709_1_MG_7D2_notaz_36h	ERS27954685	Metagenomic	Co-culture	Co-culture sample at 36h, RC start pH 8, strains K12 CMY and K12 CMYCTX No ceftazidime, (Figure S 8)
DTU_2025_1038710_1_MG_8D2_notaz_48h	ERS27954686	Metagenomic	Co-culture	Co-culture sample at 48h, RC start pH 8, strains K12 CMY and K12 CMYCTX No ceftazidime, (Figure S 8)
DTU_2025_1038711_1_MG_1C3_notaz_0h	ERS27954687	Metagenomic	Co-culture	Co-culture sample at 0h, RC start pH 5, strains K12 CTX-M and K12 CMY No ceftazidime, (Figure S 8)
DTU_2025_1038712_1_MG_2C3_notaz_3h	ERS27954688	Metagenomic	Co-culture	Co-culture sample at 3h, RC start pH 5, strains K12 CTX-M and K12 CMY No ceftazidime, (Figure S 8)
DTU_2025_1038713_1_MG_3C3_notaz_6h	ERS27954689	Metagenomic	Co-culture	Co-culture sample at 6h, RC start pH 5, strains K12 CTX-M and K12 CMY No ceftazidime, (Figure S 8)
DTU_2025_1038714_1_MG_4C3_notaz_9h	ERS27954690	Metagenomic	Co-culture	Co-culture sample at 9h, RC start pH 5, strains K12 CTX-M and K12 CMY No ceftazidime, (Figure S 8)
DTU_2025_1038715_1_MG_5C3_notaz_12h	ERS27954691	Metagenomic	Co-culture	Co-culture sample at 12h, RC start pH 5, strains K12 CTX-M and K12 CMY No ceftazidime, (Figure S 8)
DTU_2025_1038716_1_MG_6C3_notaz_24h	ERS27954692	Metagenomic	Co-culture	Co-culture sample at 24h, RC start pH 5, strains K12 CTX-M and K12 CMY No ceftazidime, (Figure S 8)
DTU_2025_1038717_1_MG_7C3_notaz_36h	ERS27954693	Metagenomic	Co-culture	Co-culture sample at 36h, RC start pH 5, strains K12 CTX-M and K12 CMY No ceftazidime, (Figure S 8)
DTU_2025_1038718_1_MG_8C3_notaz_48h	ERS27954694	Metagenomic	Co-culture	Co-culture sample at 48h, RC start pH 5, strains K12 CTX-M and K12 CMY No ceftazidime, (Figure S 8)
DTU_2025_1038719_1_MG_1D3_notaz_0h	ERS27954695	Metagenomic	Co-culture	Co-culture sample at 0h, RC start pH 8, strains K12 CTX-M and K12 CMY No ceftazidime, (Figure S 8)
DTU_2025_1038720_1_MG_2D3_notaz_3h	ERS27954696	Metagenomic	Co-culture	Co-culture sample at 3h, RC start pH 8, strains K12 CTX-M and K12 CMY No ceftazidime, (Figure S 8)
DTU_2025_1038721_1_MG_3D3_notaz_6h	ERS27954697	Metagenomic	Co-culture	Co-culture sample at 6h, RC start pH 8, strains K12 CTX-M and K12 CMY No ceftazidime, (Figure S 8)
DTU_2025_1038722_1_MG_4D3_notaz_9h	ERS27954698	Metagenomic	Co-culture	Co-culture sample at 9h, RC start pH 8, strains K12 CTX-M and K12 CMY No ceftazidime, (Figure S 8)
DTU_2025_1038723_1_MG_5D3_notaz_12h	ERS27954699	Metagenomic	Co-culture	Co-culture sample at 12h, RC start pH 8, strains K12 CTX-M and K12 CMY No ceftazidime, (Figure S 8)
DTU_2025_1038724_1_MG_6D3_notaz_24h	ERS27954700	Metagenomic	Co-culture	Co-culture sample at 24h, RC start pH 8, strains K12 CTX-M and K12 CMY No ceftazidime, (Figure S 8)
DTU_2025_1038725_1_MG_7D3_notaz_36h	ERS27954701	Metagenomic	Co-culture	Co-culture sample at 36h, RC start pH 8, strains K12 CTX-M and K12 CMY No ceftazidime, (Figure S 8)
DTU_2025_1038726_1_MG_8D3_notaz_48h	ERS27954702	Metagenomic	Co-culture	Co-culture sample at 48h, RC start pH 8, strains K12 CTX-M and K12 CMY No ceftazidime, (Figure S 8)
