# Supplementary material for: Contrasting pH optima of β-lactamases CTX-M and CMY influence Escherichia coli fitness and resistance ecology
Source: Appl Environ Microbiol. 2025 Dec 29;92(1):e01775-25. doi: 10.1128/aem.01775-25 (PMC12863049; doi:10.1128/aem.01775-25)
Supplement: Supplemental material — Figures S1 to S10; Tables S1 and S2. [file aem.01775-25-s0004.pdf]

Supplementary data

Strain construction schematic & verification

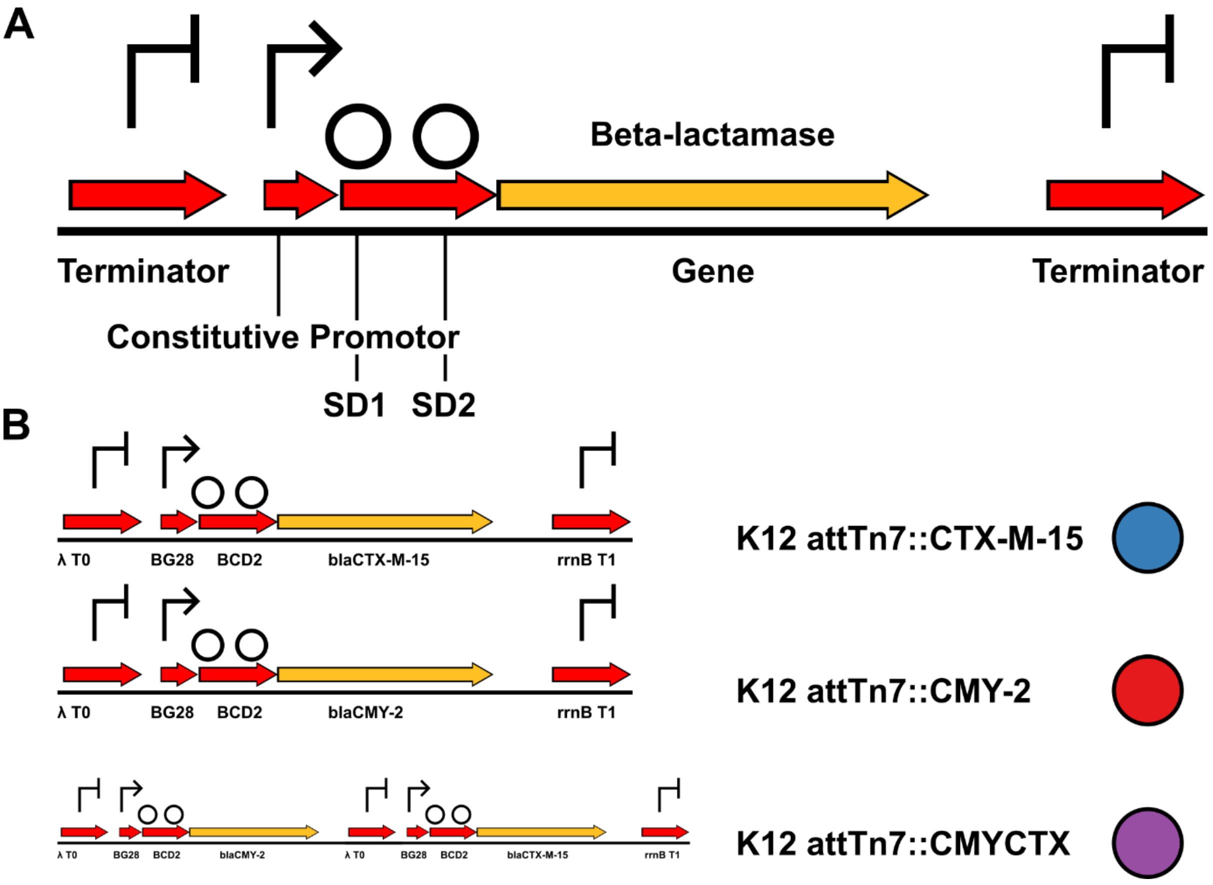

Figure S 1 β-lactamase expression constructs. A) A schematic overview of elements in the genetic constructs, non-coding regulatory elements are shown as red arrows, while coding sequences are shown as yellow arrows. B) Specific genetic elements integrated into E. coli K12 at the chromosomal attTn7 site upstream of glmS and colour scheme used for each strain in the rest of the article. Abbreviations: Shine-Dalgarno sequence (SD), β-lactamase (bla), attachment site (att).

To verify chromosomal integration and integrity of constructs in our strains, we sequenced and assembled genomes for each strain and used BLAST alignment to assess this (materials and methods). We found no SNPs within the integrated constructs, verifying the integrity of each construct.

Filamentous growth observed for K12-CTX when grown at high concentrations of ceftazidime at pH 5

Filamentous growth has previously been reported for *E. coli* K12 under pH stress, β-lactam exposure, and during urinary tract infections (UTI)(1–3), however the combination of the two (pH stress and β-lactam exposure) has not. Presumably, this is because morphological changes have primarily been studied in susceptible bacteria, where researchers have worked to elucidate drug efficacy and mechanism of action. Here we show that a specific resistance mechanism CTX-M-15, which phenotypically is most active (highest MIC (Figure 1), highest catalytic efficacy (table 1)) under acidic conditions, enables supra-MIC (>4x MIC, we tested this up to 256µg/ml ceftazidime and we still saw filamentous growth at pH 5) persistence toward ceftazidime by filamentation (Figure S 2). Based on this, it might be inadvisable to treat UTI's harbouring CTX-M-15 with cephalosporins (ceftazidime & cefotaxime), on the other hand if an urinary alkalinizer is utilized to increase urine pH (with e.g. potassium citrate or sodium bicarbonate) this infection may be treatable with cephalosporins.

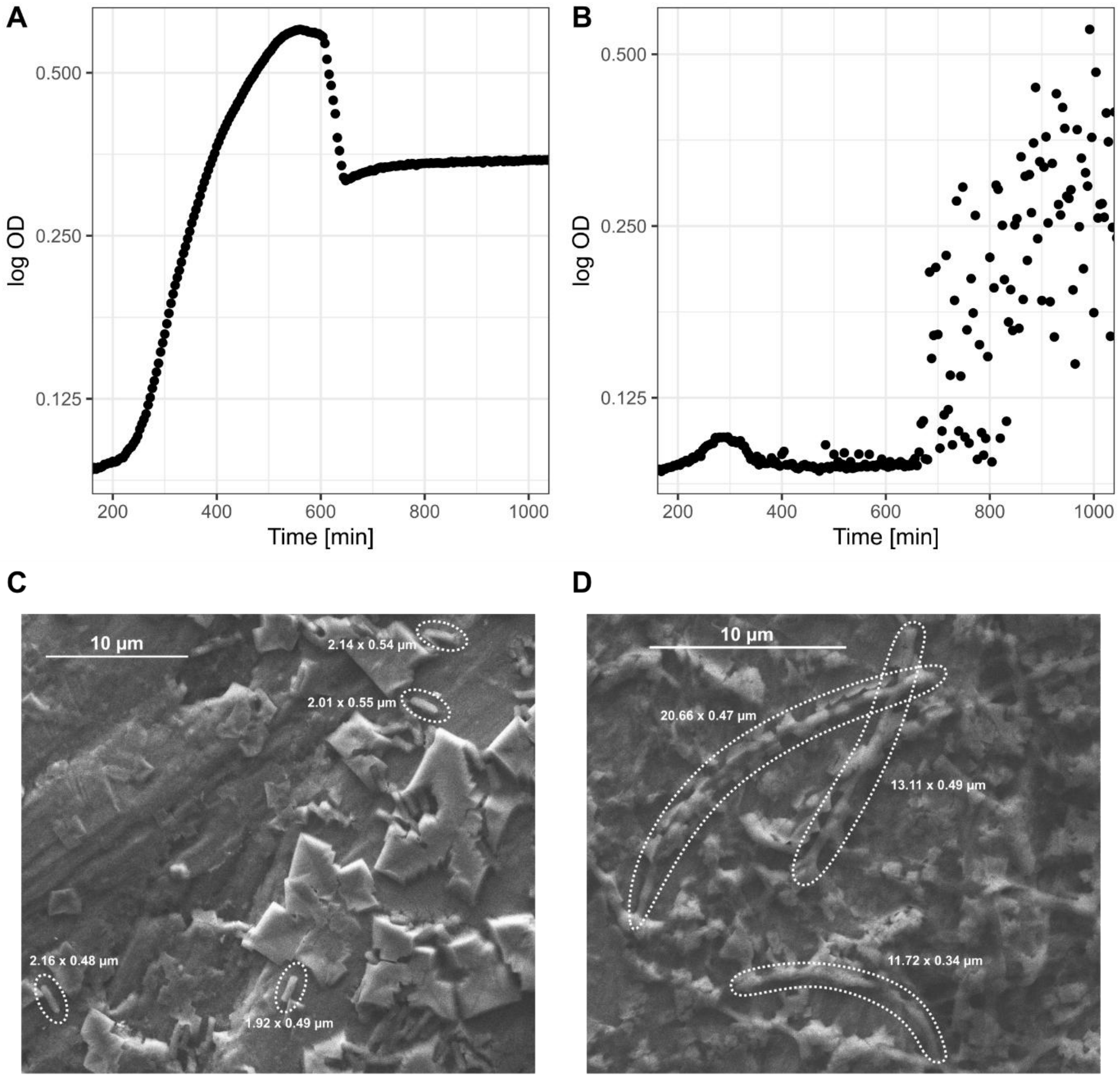

Figure S 2 A) A characteristic kinetic growth curve of K12 CTX-M grown in MHB at pH 5 without added antibiotic. B) An unusual kinetic growth curve of K12 CTX-M grown in MHB at pH 5 at 64µg/ml ceftazidime. The highly fluctuating OD measurements indicate dense objects which move in and out of the light path. C) Cryo-EM image of K12 CTX-M grown in MHB pH 5. Cells have been outlined with dotted white circles and their dimensions in µm are shown next to each cell. D) Cryo-EM image of K12 CTX-M grown in MHB pH 5 supplemented with 64 µg/ml ceftazidime. Cells primarily exhibit a filamentous morphology under these conditions, reaching up to 20 µm in length. This phenotype (filamentation) is also associated with immune evasion during UTIs and highlights the potential importance of pH management during antibiotic therapy.

List of primers

Table S 1: List of primers used in this study, each primer is given in the 5'-3' direction

Name	Sequence	Purpose

Pr3 tn7 fw NcoI	TGCGGCCGCACTGCCACTC ATCGCAGTCTA	Preparation of pUC18R6KT-mini-Tn7T-Gm for integration of oligo (BG28 BCD2)
Pr4 tn7 rev NotI	GTGCCATGGTATTCGCCTGG GGTAATGACTCTC	Preparation of pUC18R6KT-mini-Tn7T-Gm for integration of oligo (BG28 BCD2)
Pr5 CTXM15 gibs fw	ATCTTAATCATGCTAAGGA GGTTTTCTAATGGTTAAAAA ATCACTGCGCC	PCR of CTXM15 gene with homology tails to mini-tn7 vector
Pr6 CTXM15 gibs rev	AGGCGAATACCATGGCACC GCCTGGAATCTTACAAACC GTCGGTGACGAT	PCR of CTXM15 gene with homology tails to mini-tn7 vector
Pr7 CMY-2 gibs fw	ATCTTAATCATGCTAAGGA GGTTTTCTAATGATGAAAA AATCGTTATGC	PCR of CMY2 gene with homology tails to mini-tn7 vector
Pr8 CMY-2 gibs rev	AGGCGAATACCATGGCACC GCCTGGAATCTTTATTGCAG CTTTTCAAGAA	PCR of CMY2 gene with homology tails to mini-tn7 vector
Pr9 mini-tn7 seq fw	TATTAAAGAGGGGCGTGGG	Sequencing of genes integrated into mini-tn7 vector
Pr10 mini-tn7 seq rev	AGAGCGTTCACCGACAAA	Sequencing of genes integrated into mini-tn7 vector
Pr23 mini-tn7 rev2	CAAGCTAGAGAGTCATTAC C	For double gene construct (pMA1 CMYCTX)
Pr45 BCD2 rev	ATTAGAAAACCTCCTTAGC A	PCR Linearization of pMA1 BG28 vector for gibson assembly
Pr69 pMA1 linear fw	AGATTCCAGGCGGTGCCAT G	PCR Linearization of pMA1 BG28 vector for gibson assembly
Pr87 NcoI pr9v2 fw	TTATCCATGGTATTAAAGA GGGGCGTGG	For double gene construct (pMA1 CMYCTX)

Quantification of βlactams with spectroscopy

Since we chose to measure the rate of β-lactam hydrolysis with a plate-reader we could not utilize empirically determined molar absorption coefficients to determine the concentration of samples (as the path length is dependent on sample volume, which we did not measure). Therefore, we first determined the spectrum for nitrocefin in hydrolysed and unhydrolyzed

form (300-800nm hydrolysed 1 minute with 54 nM CTX-M-15 or 43nM CMY-2 (Figure S 3 A)). This let us determine the wavelength with the largest difference between absorbance of hydrolysed and unhydrolysed substrates at different wavelengths (both 390 and 490nm for nitrocefin; 490 for hydrolysed form, 390 for unhydrolyzed).

For nitrocefin we were able to distinguish between the hydrolysed and the unhydrolyzed form at 390 and 490nm, respectively. For each sample with and without enzyme added, we measured absorbance at both wavelengths. The amount of hydrolysed nitrocefin is quantified using the absorbance of the untreated nitrocefin sample measured at 390 and 490 nm, where the absorbance at 390nm equals full hydrolysis and the absorbance at 490nm equals no hydrolysis (figure S 3 A). This is based on our observation that the absorbance at 490nm of fully hydrolysed nitrocefin is equal to the absorbance of unhydrolyzed nitrocefin at 390nm, at pH 7 (n=2). This is not completely accurate for pH 5 or 9, so for quantifying nitrocefin hydrolysis, pH 5 absorbance at 390nm was divided by 1.056, and pH 9 absorbance at 390nm was divided by 0.986 to correct for this.

To quantify the amount of unhydrolyzed nitrocefin we use a linear regression between a solvent control and a known amount of nitrocefin at 390nm (figure S 3 B) for each pH and substrate concentration assayed.

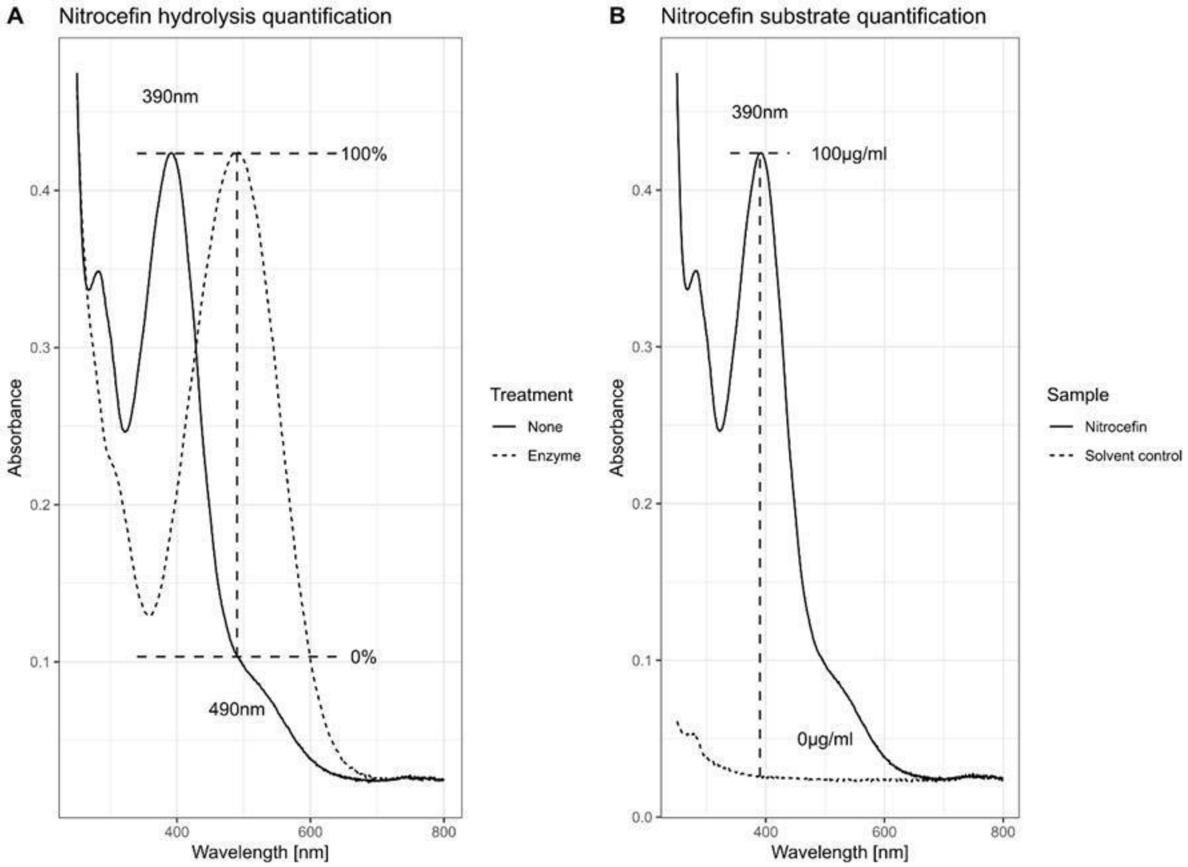

Figure S 3 Spectroscopic quantification of hydrolysed (A) and unhydrolyzed nitrocefin (B). A) Spectra of nitrocefin (solid line) and hydrolysed nitrocefin (dashed line). B) Spectra of nitrocefin (solid line) and solvent control (dashed line) used to quantify unhydrolyzed nitrocefin. Spectra are representative of 2 replicates.

Growth kinetics of strains

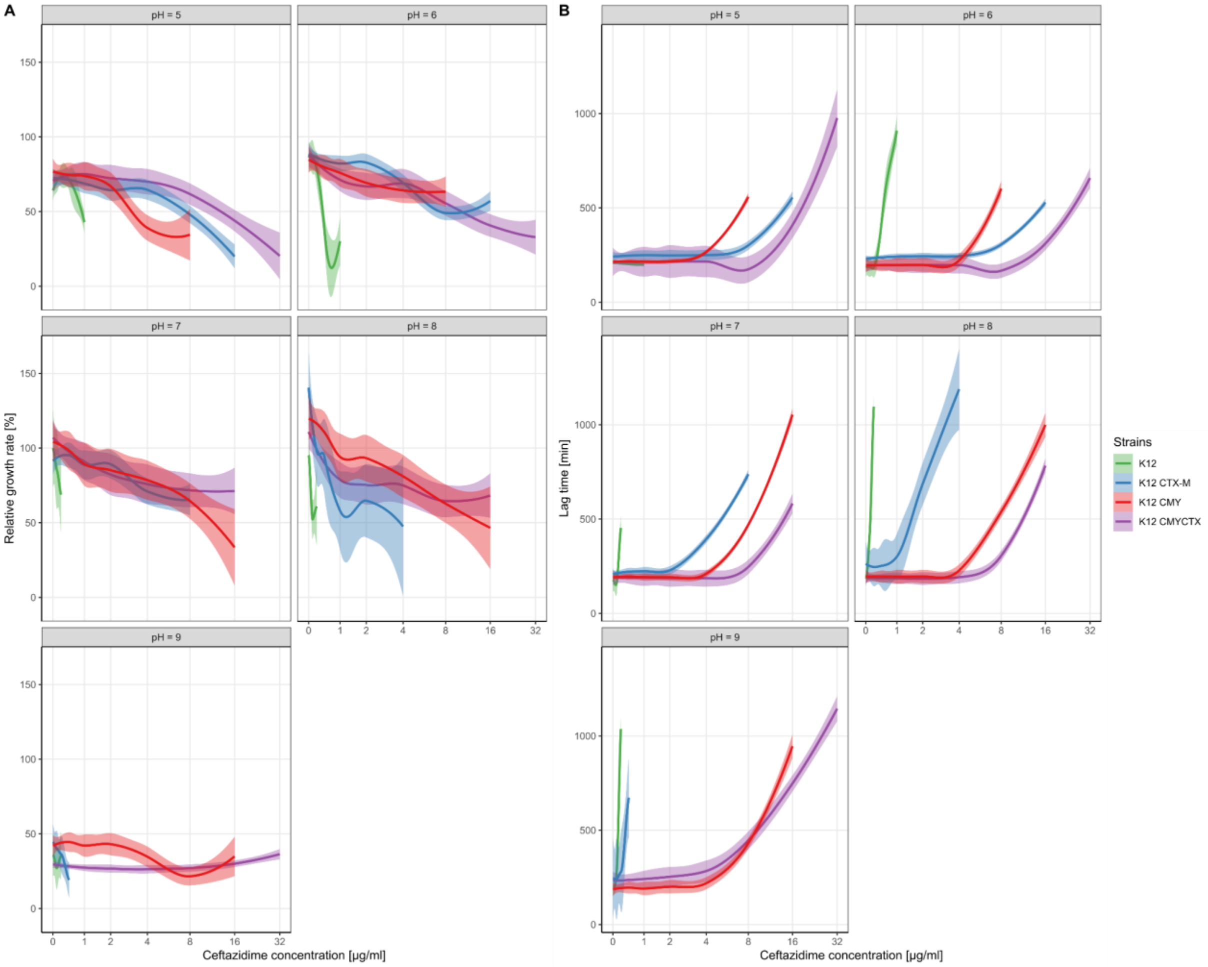

Figure S 4 Result of growth rate assays summarising growth rates A) and lag times B) with plots showing the smoothed conditional means for each strain along with the 95% confidence interval (lighter shaded area around the lines). Each condition was replicated 3 times (pH, strain, ceftazidime concentrations 0-32 µg/ml). A) The relative growth rate of each strain has been normalized to the mean growth rate of the wild-type K12 at pH 7. Growth rates (ordinate) were assayed in pH's 5, 6, 7, 8, 9 (different panels) at different concentrations of ceftazidime (abscissa). B) The lag time (time until exponential growth) of strains under each condition was determined (similar to A, except the y-axis shows the lag time).

Ceftazidime CTX-M-15 hydrolysis rates

To determine which wavelength to measure, we determined the spectrum of hydrolysed and unhydrolysed ceftazidime (between 200-300nm for ceftazidime hydrolysed for 4 hours at 37 C with 76 nM CTX-M-15). We determined that the wavelength with the largest difference in absorbance between the hydrolysed and the unhydrolysed substrate was 266 nm for ceftazidime.

To quantify ceftazidime, we measured absorbance of a dilution row at 266nm (460-1 µg/ml). Using linear regression, we found a good linear relationship between absorbance and concentration between 250 and 4 µg/ml, which was used to quantify unhydrolyzed ceftazidime (Figure S 5 B). While untreated ceftazidime has a peak at 257 nm, we find that the fit between absorbance and concentration is worse at this wavelength ($R^2 = 0.9855$).

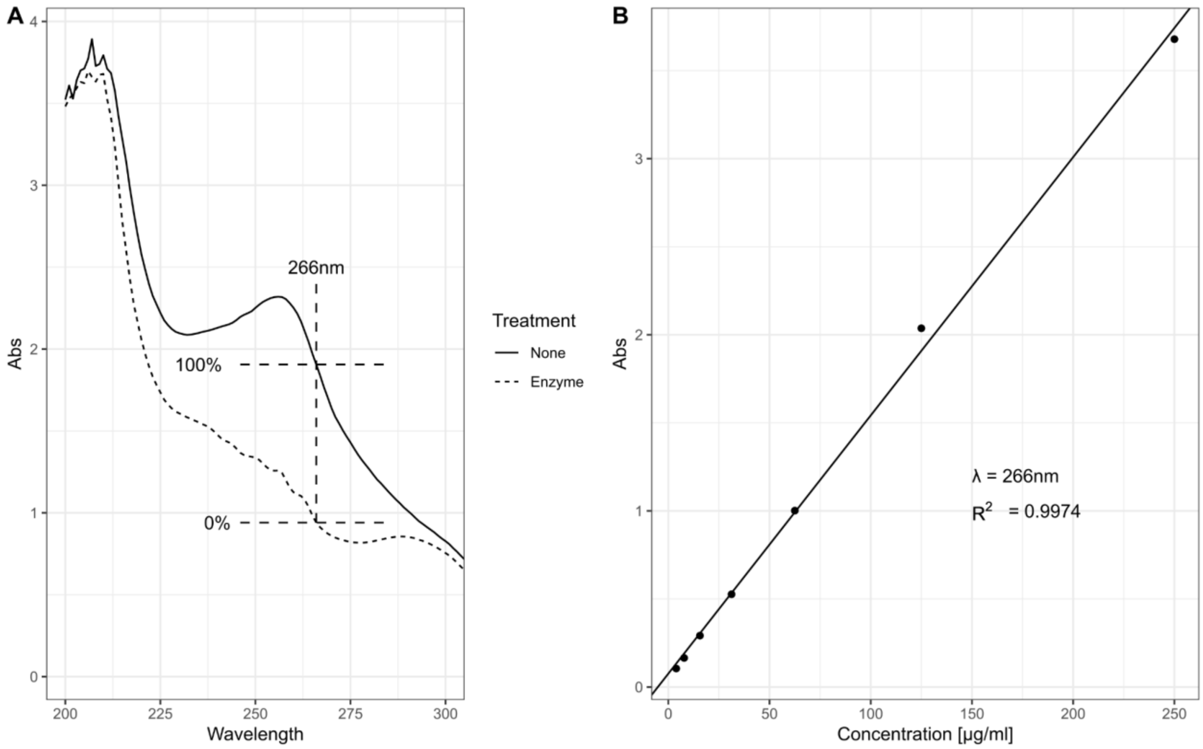

Figure S 5 Spectroscopic quantification of hydrolysed and unhydrolyzed ceftazidime. A) Spectra of ceftazidime (solid line) and hydrolysed ceftazidime (dashed line). The vertical dashed line marks 266nm in the spectra. To quantify hydrolysis B) Linear relationship between absorbance and concentration of unhydrolyzed ceftazidime at 266 nm

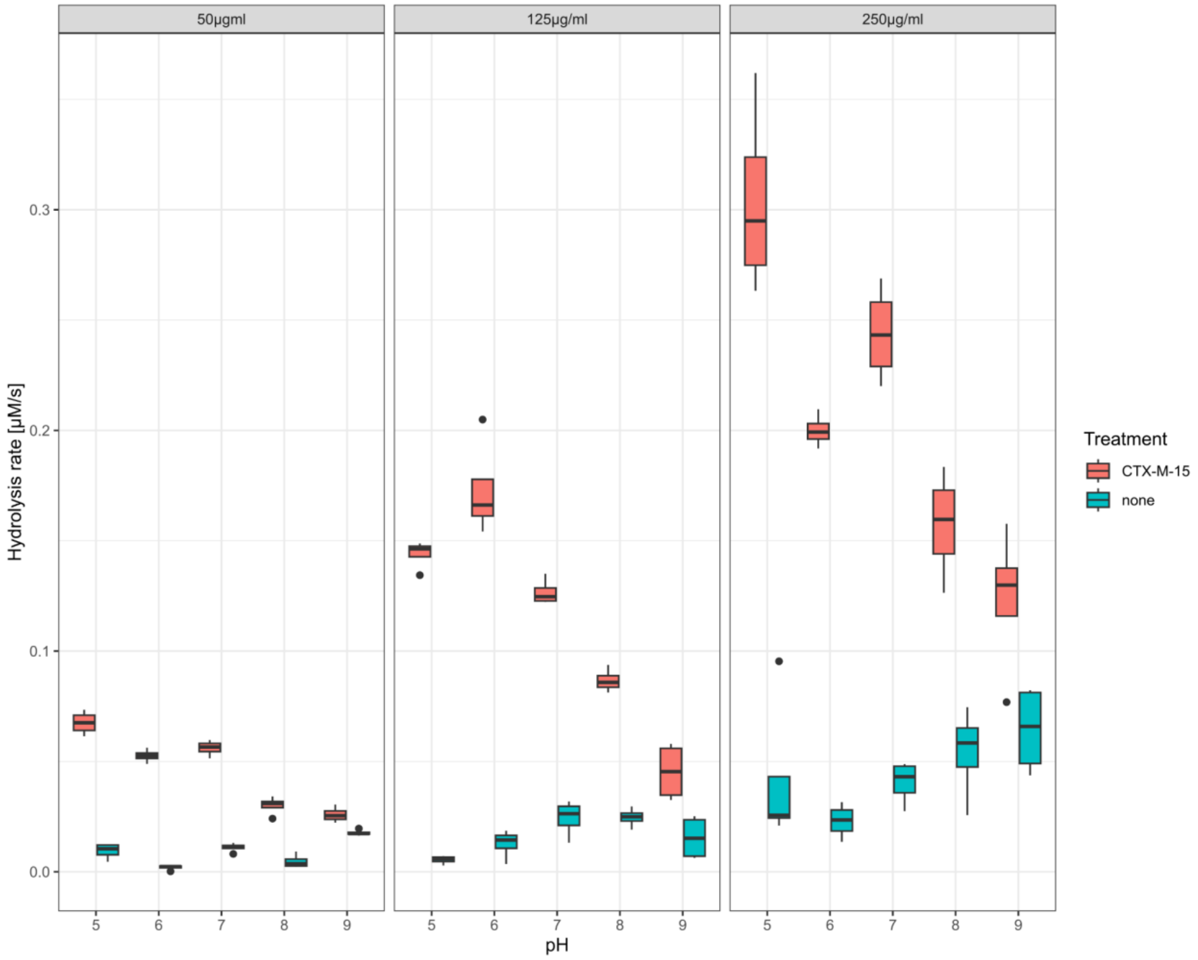

Figure S 6 Ceftazidime hydrolysis rates with CTX-M-15. Different initial concentrations of ceftazidime (top panels of plots) were incubated at 37°C with or without 50.05 nM CTX-M-15 at the indicated pH values. The hydrolysis was followed by measuring absorbance at 266nm. Each condition was replicated 4 times. Outliers are shown as circles, using the default ggplot2 boxplot method.

Co-cultures with no added ceftazidime section

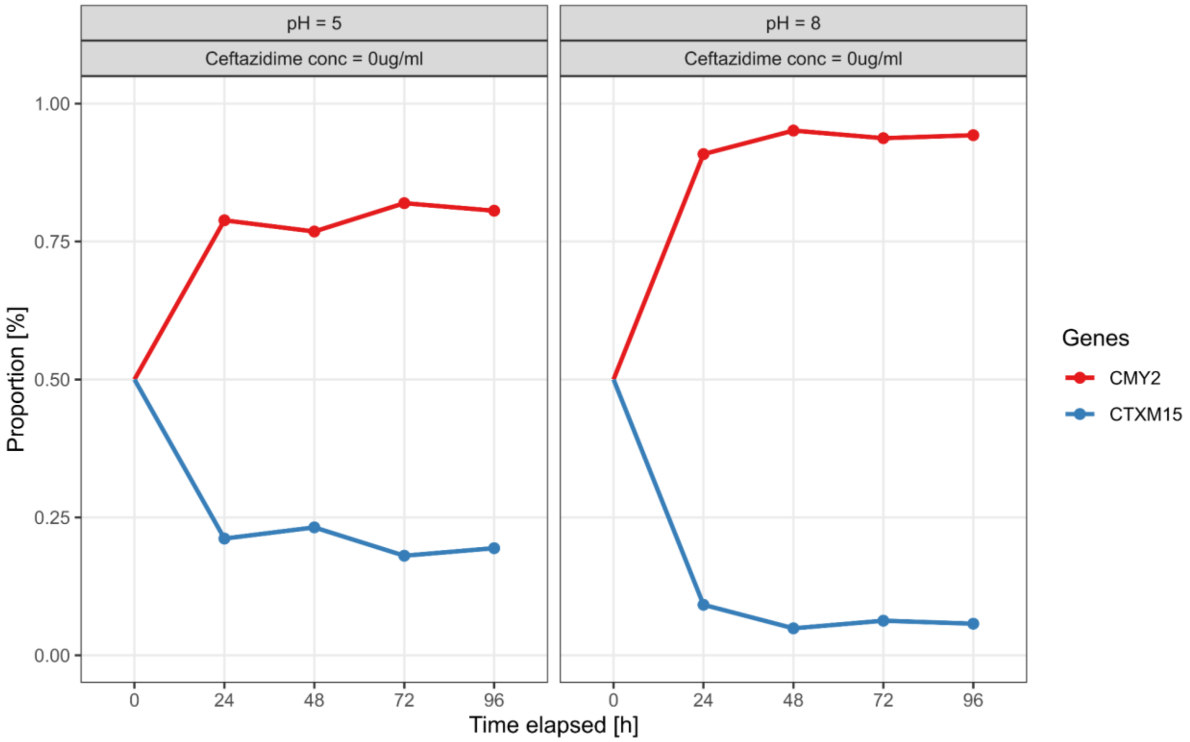

Figure S 7 Co-cultures of K12 CTX-M and K12 CMY at constant pH 5 or 8 (top of plot) with no added ceftazidime. Co-cultures were passaged to fresh media after sampling every 24 hours. The plot shows the relative proportion of strains in the cultures. Regardless of pH, K12 CMY becomes the most abundant strain after 24 hours.

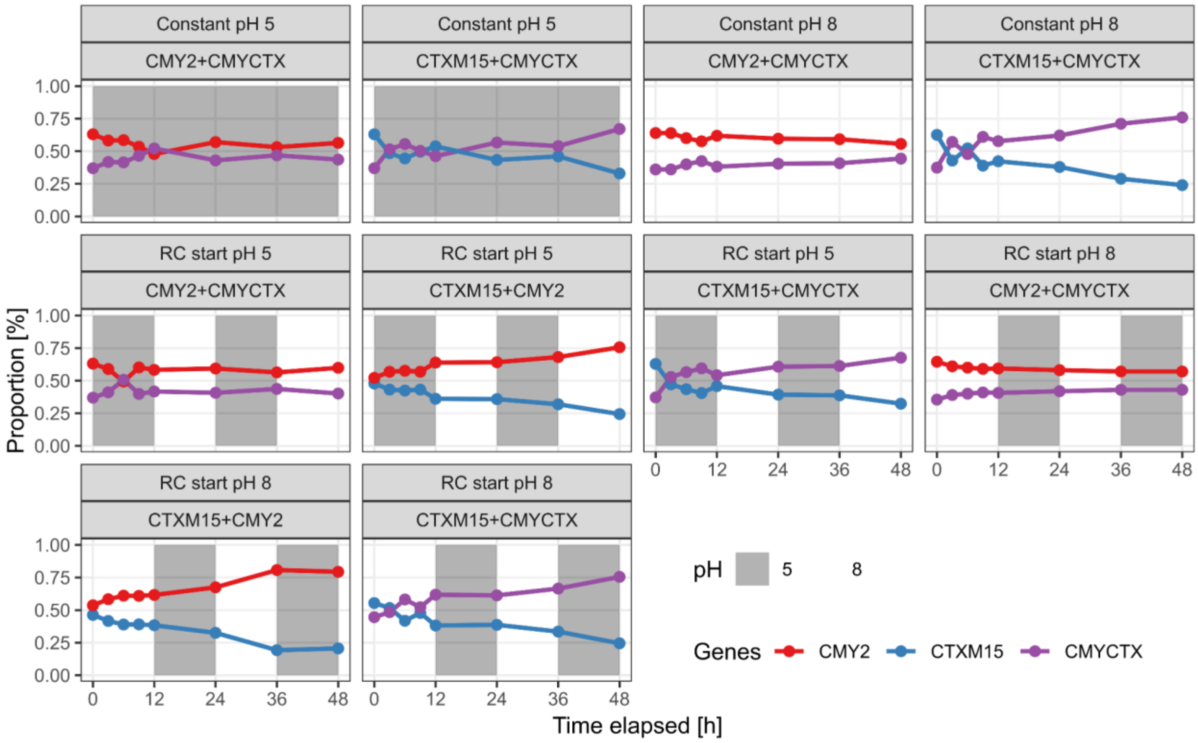

Figure S 8 Co-cultures of strains at constant or rapidly changing (RC) pH in media with no added ceftazidime. The relative proportion of each strain has been shown in each plot, while the conditions of each co-culture has been indicated in panels above plots. Solid circles

indicate samples where the corresponding strain could be detected using sequencing (all samples).

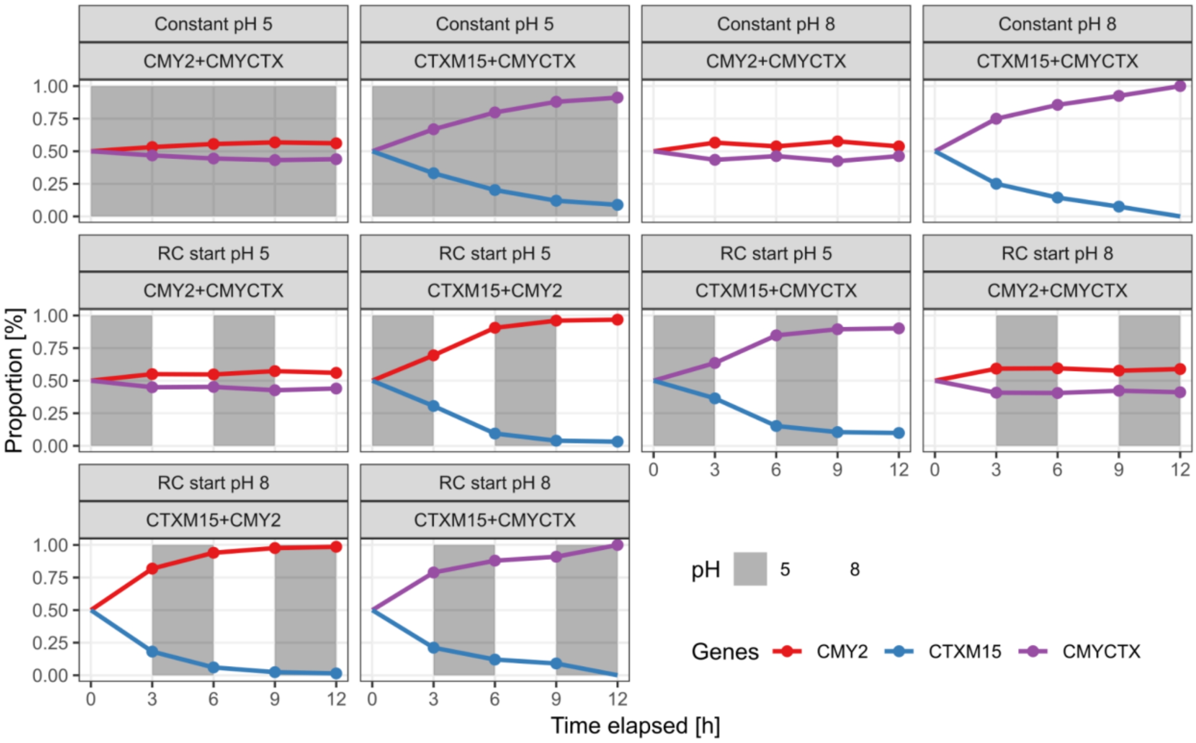

Figure S 9 Co-cultures of strains at constant or rapidly changing (RC) pH in media with no added ceftazidime. The relative proportion of each strain has been shown in each plot, while the conditions of each co-culture has been indicated in panels above plots. Solid circles indicate samples where the corresponding strain could be detected using sequencing. Strains below the detection limit are set to 0 proportion. We added an initial 0.5 proportion at t = 0, however we did not sample at this time point.

Equations visualised

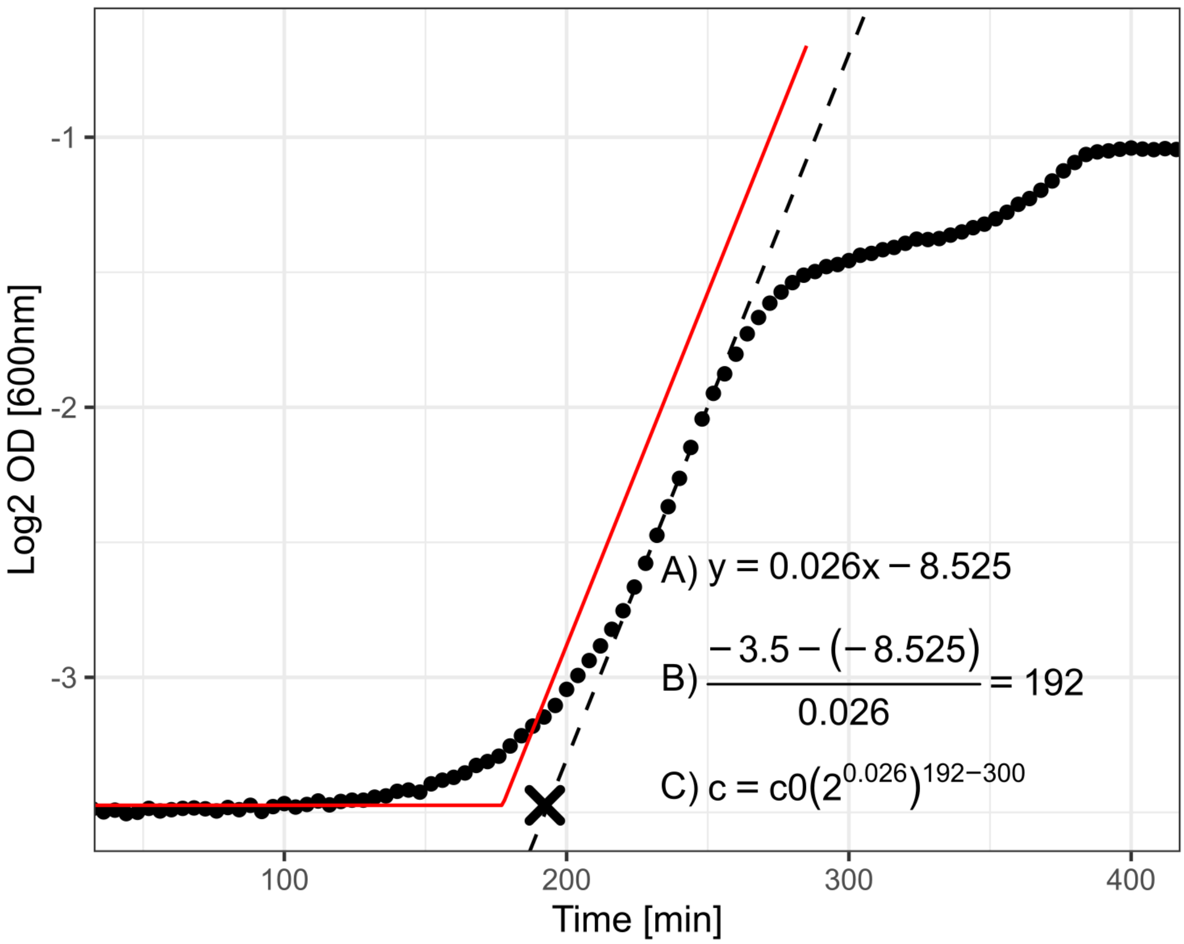

Figure S 10 Equations visualised for a representative growth curve. A bacterial culture was grown in a microtiter plate and every 4 minutes we measured the absorbance at 600 nm (black points). A) When the culture enters exponential growth phase (approx 210-220 minutes), we use linear regression to determine the growth rate of the strain at a given condition according to y=ax+b. (dashed black line) B) To estimate the lag time (time until exponential phase), we isolate x (equation 1) in y=ax+b, this time is marked in the plot as X. C) For the purposes of fitness calculations, we use the equation for exponential growth (eq 2) to simulate growth (shown as a red line and shifted -15 minutes for the sake of the illustration as it would otherwise overlap with the black dashed line), using the rate from A) and the lagtime from B).

Detailed MIC response of clinical isolates to pH changes

Supplementary table S2: Ceftazidime MIC of clinical isolates. The mean ceftazidime MIC at the specified pH ± the standard deviation has been shown in the table for each isolate.

Isolate	Gene	pH 5	pH 6	pH 7	pH 8	pH 9
TWIW_02_DEU_MAG_BI_061	CMY-2	8±0	16±0	32±0	32±0	32±0
TWIW_02_DEN_HVI_BM_056	CMY-2	32±0	64±0	106.7±37	128±0	256±0
TWIW_02_CHE_ZUR_BX_012	CMY-2	4±0	10.7±4.6	16±0	32±0	128±0
TWIW_01_GHA_SEK_008	CTX-M-15	106.7±37	106.7±37	106.7±37	53.3±18.5	8±0
TWIW_02_NGA_ILE_BN_040	CTX-M-15	53.3±18.5	42.7±18.5	10.7±4.6	4±0	1±0
TWIW_02_ALB_TIR_AI_026	CTX-M-15	85.3±37	128±0	64±0	42.7±18.5	8±0
TWIW_02_KAZ_ALM_CD_036	CTX-M-15	53.3±18.5	128±110.9	32±0	21.3±9.2	4±0
2010_60_1061_1	CMY-2	10.7±4.6	16±0	21.3±9.2	32±0	64±0
2010_60_5498_4	CMY-2	6.7±2.3	8±0	13.3±4.6	8±0	26.7±9.2
2010_60_5499_19	CMY-2	8±0	13.3±4.6	16±0	21.3±9.2	42.7±18.5
2010_60_7075_19	CMY-2	6.7±2.3	6.7±2.3	8±0	10.7±4.6	16±0
2010_60_7077_57	CMY-2	8±0	13.3±4.6	16±0	16±0	32±0
2010_60_7077_7	CMY-2	8±0	13.3±4.6	8±0	16±0	37.3±24.4
2028_7	CMY-2	8±0	16±0	21.3±9.2	32±0	64±0
2028_8	CMY-2	8±0	16±0	16±0	32±0	53.3±18.5
2067_2	CMY-2	8±0	16±0	16±0	32±0	64±0

2115_5	CMY-2	5.3±2.3	8±0	13.3±4.6	16±0	32±0
TWIW_02_LTU_KAU_BG_025	CTX-M-15	32±0	32±0	16±0	8±0	3.3±1.2
TWIW_01_PAK_PES_036	CTX-M-15	3.3±1.2	2±0	1.7±0.6	1±0	0.7±0.3
TWIW_02_NGA_ABU_AD_008	CTX-M-15	32±0	32±0	21.3±9.2	8±0	2.7±1.2
TWIW_02_PAK_PES_AA_062	CTX-M-15	32±0	26.7±9.2	16±0	8±0	2.7±1.2
TWIW_01_CZE_PRA_051	CTX-M-15	64±0	64±0	32±0	13.3±4.6	3.3±1.2
TWIW_02_PAK_PES_AA_013	CTX-M-15	5.3±2.3	4±3.5	2±0	0.7±0.3	0.4±0.1
TWIW_02_CHE_ZUR_BX_047	CTX-M-15	16±0	8±0	4±0	2±0	1±0
TWIW_01_NA_ABU_020A	CTX-M-15	53.3±18.5	21.3±9.2	16±0	13.3±4.6	3.3±1.2
TWIW_02_FRA_LIL_AK_033	CTX-M-15	13.3±4.6	16±0	8±0	5.3±2.3	0.5±0.4
TWIW_01_TUR_ORT_046	CTX-M-15	256±0	128±0	53.3±18.5	32±0	10.7±4.6

References

1. Justice SS, Hunstad DA, Seed PC, Hultgren SJ. 2006. Filamentation by Escherichia coli subverts innate defenses during urinary tract infection. Proc Natl Acad Sci U S A 103:19884–19889.

2. Justice SS, Hunstad DA, Cegelski L, Hultgren SJ. 2008. Morphological plasticity as a bacterial survival strategy. Nat Rev Microbiol 6:162–168.

3. Cushnie TPT, O’Driscoll NH, Lamb AJ. 2016. Morphological and ultrastructural changes in bacterial cells as an indicator of antibacterial mechanism of action. Cell Mol Life Sci 73:4471–4492.
