# Supplementary material for: Contrasting pH optima of β-lactamases CTX-M and CMY influence Escherichia coli fitness and resistance ecology
Source: Appl Environ Microbiol. 2025 Dec 29;92(1):e01775-25. doi: 10.1128/aem.01775-25 (PMC12863049; doi:10.1128/aem.01775-25)
Supplement: Supplemental legends — Legends supplemental HTML files. [file aem.01775-25-s0005.pdf]

Legends for Supplemental Material - "3D model from Figure 3" and "3D model form Figure 3, without the CMYCTX construct".

Interactive fitness landscape figures 1 and 2 (html) representing figure 3A and 3B, respectively. Fitness landscape of β-lactamase expressing strains, showing the most fit strain at a given ceftazidime concentration and pH. A): At sub-MIC concentration (0.125-4 µg/ml) of ceftazidime, K12 CMY is fittest. On the other hand, K12 CTX-M appears to be fittest in a niche at acidic pH above 4 µg/ml ceftazidime. However, with increasing antibiotic stress K12 CMYCTX starts to take over across all pHs assayed, illustrating how harbouring multiple different β-lactamases can be beneficial to the success and survival of a strain. B) same as A but without K12 CMYCTX.
